# Prevalence of Oropharyngeal Dysphagia in Adults in Different Healthcare Settings: A Systematic Review and Meta-analyses

**DOI:** 10.1007/s00455-022-10465-x

**Published:** 2022-05-31

**Authors:** Maribeth Caya Rivelsrud, Lena Hartelius, Liza Bergström, Marianne Løvstad, Renée Speyer

**Affiliations:** 1 Department of Research, Sunnaas Rehabilitation Hospital, Bjørnemyr, Norway; 2 Institute of Neuroscience and Physiology, Speech and Language Pathology Unit, Sahlgrenska Academy, Gothenburg University, Gothenburg, Sweden; 3 Skaraborgs Hospital, Skövde, Sweden; 4 Speech Pathology, Division of Neurology, Department of Clinical Sciences, Karolinska Institutet, Danderyd University Hospital, Stockholm, Sweden; 5 REMEO Stockholm, Stockholm, Sweden; 6 Department of Psychology, University of Oslo, Oslo, Norway; 7 Department of Special Needs Education, University of Oslo, Oslo, Norway; 8 Department of Otorhinolaryngology and Head and Neck Surgery, Leiden University Medical Centre, Leiden, the Netherlands; 9 Curtin School of Allied Health, Faculty of Health Sciences, Curtin University, Perth, WA Australia

**Keywords:** Deglutition, Swallowing disorders, Hospital, Rehabilitation, Nursing home, Prevalence

## Abstract

Oropharyngeal dysphagia (OD) is prevalent in the elderly and persons with complex medical conditions, resulting in considerable medical and psychosocial consequences and reduced quality of life. Many prevalence studies regard OD in relation to age or diagnosis. Knowledge on the prevalence of OD in different healthcare settings is lacking. This systematic review aimed to estimate the prevalence of OD in adults admitted to hospitals, rehabilitation facilities, nursing homes, and palliative care facilities through meta-analyses. A systematic literature search was completed including all dates up to March 30, 2021. The methodology and reporting were based on the Preferred Reporting Items for Systematic Reviews and Meta-Analyses (PRISMA). Forty-four out of 1,956 screened articles were deemed eligible. Considerable heterogeneity in definitions of OD and type and quality of selected outcome measures were observed. Overall within-group pooled prevalence estimates for OD determined by meta-analysis were 36.5% (95% CI 29.9 − 43.6) in the hospital setting, 42.5% (95% CI 35.8 − 49.5) in the rehabilitation setting, and 50.2% (95% CI 33.3–67.2) in nursing homes. No OD prevalence data were identified for palliative care facilities. Results for between-group analyses of OD prevalence estimates in the hospital setting were non-significant for type of assessment method, diagnostic group, and type of hospital ward, but indicated significantly higher prevalence estimates in nursing homes when using screening compared to patient-report. Future research should provide OD prevalence data for palliative care, achieve consensus in OD-related terminology when performing prevalence studies, and use screening and assessments with optimal diagnostic performance and psychometric properties.

**Supplementary Information:**

The online version contains supplementary material available at 10.1007/s00455-022-10465-x.

## Introduction

Oropharyngeal dysphagia (OD) is prevalent in various neurological etiologies (e.g., stroke, traumatic brain injury, Parkinson’s disease), or as a consequence of respiratory disease or structural changes (e.g., head and neck cancer, spinal cord injury) [1–4]. OD has also been acknowledged as a geriatric syndrome [5]. Physiological changes that occur in swallowing function in older healthy adults (presbyphagia) may be worsened by age-related decline in muscle mass and strength (sarcopenia), thus exacerbating OD caused by disease (e.g., stroke, Parkinson’s disease) common to the aging population [6]. Older adults who have changes in swallowing function are often unaware that they have OD or that it is treatable [7, 8].

Serious medical consequences of OD such as malnutrition, dehydration, pneumonia, and the need for enteral nutrition contribute to increased institutionalization; increased length of hospital stay, increased hospital re-admissions, and likelihood of being discharged to rehabilitation services and nursing homes instead of home [9, 10].

The complexity of OD requires a multidimensional approach to diagnosis in order to plan individually tailored intervention [5]. Identification of OD is completed by either screening, clinical non-instrumental assessment, or instrumental assessment such as videofluoroscopic swallowing study (VFSS) [11] or fiberoptic endoscopic evaluation of swallowing (FEES) [12]. A screening is a test given to distinguish between persons at risk from those that are not at risk of OD and helps to determine the need for further clinical non-instrumental or instrumental assessment [13]. In contrast, a non-instrumental assessment of OD is a more comprehensive evaluation and may include a medical history, assessment of orofacial sensorimotor and laryngeal function, and assessment of swallowing function using foods and liquids in various volumes and consistencies, determining the phase(s) of swallowing process that are deficient. These findings aid in determining dysphagia severity, possible treatment strategies and support the need for instrumental assessment [13]. Instrumental assessments, VFSS and FEES, are noted as being preferred instrumental assessments for OD in the literature although no international consensus exists on which visuo-perceptual measure to use when evaluating the radiological or endoscopic recordings of swallowing [14]. The use of patient-reported outcome measures (PROMs) provides a subjective assessment of the patients’ perspectives on the burden of living with OD [13].

Prevalence research is important as it reflects the burden of a disease or condition in a population at a particular time period. A systematic review by Kertscher and colleagues found that prevalence data on oropharyngeal dysphagia for the general population varied between 2.3 and 16% [15]. However, much of the available research reviewing the prevalence of OD is targeted toward populations according to age or diagnosis. A systematic review and meta-analysis by Madhavan et al. revealed a prevalence of OD ranging from 5 to 72% in the community-dwelling elderly population [16]. Takizawa and colleagues reviewed a broad spectrum of disorders susceptible for OD and found a prevalence of 8–45% in relation to stroke, 11–60% in Parkinson’s disease, and 27–30% in traumatic brain injury [1]. No studies were identified for prevalence of OD associated with Alzheimer’s disease [1].

Variations in reported OD prevalence can be attributed to methodological differences such as clinical setting, how dysphagia is defined, the study population, choice of measurement tools used, and time of assessment [1]. The severity of OD varies within the course of an illness or disease and can be defined as acute or chronic, and progressive or non-progressive [17]. Thus, the timing of assessment in relation to type and onset of illness or disease can impact the accuracy of OD prevalence data. Furthermore, healthcare professionals’ knowledge of OD and their routines for identification of OD have been found to be inadequate [7, 18]. Thus, OD is under-diagnosed and under-reported [1].

Existing literature on OD prevalence in different healthcare settings is mainly found in individual prevalence studies. There are systematic reviews on OD prevalence in adults with different diagnosis [1, 19, 20] and the community-dwelling elderly [16], but there is currently no overview of the prevalence of OD in the hospital, rehabilitation, nursing home, or palliative healthcare settings. Evidence about the scope of OD in adults in different healthcare settings will provide insight on the impact of OD in different settings. This evidence will increase healthcare professionals’ awareness of the likelihood of patients/residents presenting with OD and aid policy makers when assessing the allocation of interdisciplinary resources to meet the needs of persons with OD. The aim of this systematic review is thus to determine prevalence estimates of OD in adults admitted to hospitals, rehabilitation, nursing homes, and palliative care facilities using meta-analyses.

## Materials and Methods

The protocol for this systematic review and meta-analysis was registered with the international prospective register of systematic reviews (PROSPERO; registration number CRD42019134585) in August 2019. The methodology and reporting of results is based on the Preferred Reporting Items for Systematic Reviews and Meta-Analyses (PRISMA), which aims to ensure complete and transparent reporting of systematic reviews and meta-analyses [21].

### Eligibility Criteria

Studies were considered eligible for inclusion for this systematic review if they (1) reported on persons with oropharyngeal dysphagia (OD), (2) provided data on prevalence, frequency, or incidence, (3) described adult populations (≥ 18 years of age) ,and, (4) referred to healthcare settings (including hospital, rehabilitation, nursing home, or palliative care facilities).

Study inclusion was not limited by study design; however, only peer-reviewed original studies in English were included, and thus conference abstracts, review articles, case reports, student dissertations, and editorials were excluded. In order to minimize selection bias, prevalence estimates based on preselected groups (e.g., selected numbers of adults who already failed any previous form of OD screening or adults selected by specific comorbidity or surgical procedure) were excluded. In order to improve the level of precision in the prevalence estimates, studies with sample sizes below 30 participants were excluded.

### Search Strategy and Study Selection

A literature search was completed on March 30, 2021, in two electronic databases: Embase and PubMed. Terms related to dysphagia, clinical settings (hospital, rehabilitation, nursing home, palliative care), and prevalence were entered into each electronic database to retrieve all relevant subject headings (i.e., MeSH and Thesaurus terms). In addition, free text terms were included in combination with field searches (i.e., Title/Abstract) and truncation (i.e., wildcards). Subject headings and free text terms were combined using Boolean operators to either expand searches (i.e., Boolean operator “OR”) or to restrict and combine searches (i.e., Boolean operator “AND”). All publication dates up to the search date were included. Search strategies are presented in Table 1. Two independent reviewers completed a structured assessment for eligibility. Prior to independently screening all titles and abstracts, the reviewers completed two training sessions, with a sample of 100 abstracts, in order to establish a consensus on the interpretation of the eligibility criteria. Both reviewers independently screened all titles and abstracts. The same two independent reviewers completed a full-text review of selected articles for assessment of eligibility. The reviewers also searched the references from the included articles to identify additional eligible articles. Any discrepancies of inclusion between reviewers were settled by consensus throughout the review process. When in doubt, the two main reviewers conferred with a third reviewer whom is experienced in PRISMA methodology.

**Table 1:** Search strategies per literature database

Database and search terms	Number of records
*Embase*: (Swallowing/OR Dysphagia/) AND (Prevalence/OR Incidence/OR Epidemiology/) AND (rehabilitation/OR rehabilitation care/OR rehabilitation center/OR rehabilitation medicine/OR rehabilitation nursing/OR nursing home/OR hospital/OR hospice/OR hospice care/OR hospice nursing/) OR ((swallow OR dysphag* OR deglut*).ab,ti. AND (Prevalence* OR incidence*).ab,ti. AND (((nursing AND home) OR (nursing AND homes) OR rehabilitation* OR hospice* OR palliat*)ab,ti. OR (hospital OR hospitals)ti.))	898
*PubMed*: (("Deglutition"[Mesh] OR "Deglutition Disorders"[Mesh]) AND ("Prevalence"[Mesh] OR "Epidemiology"[Mesh] OR "Incidence"[Mesh]) AND ("Hospitals"[Mesh] OR "Hospital Medicine"[Mesh] OR "Hospital Mortality"[Mesh] OR "Cardiology Service, Hospital"[Mesh] OR "Physical Therapy Department, Hospital"[Mesh] OR "Outpatient Clinics, Hospital"[Mesh] OR "Occupational Therapy Department, Hospital"[Mesh] OR "Nursing Staff, Hospital"[Mesh] OR "Medical Staff, Hospital"[Mesh] OR "Hospitals, Urban"[Mesh] OR "Hospitals, Military"[Mesh] OR "Tertiary Care Centers"[Mesh] OR "Hospitals, Chronic Disease"[Mesh] OR "Secondary Care Centers"[Mesh] OR "Hospitals, Private"[Mesh] OR "Hospitals, Veterans"[Mesh] OR "Hospitals, State"[Mesh] OR "Hospitals, Special"[Mesh] OR "Hospitals, Public"[Mesh] OR "Hospitals, General"[Mesh] OR "Hospitals, Municipal"[Mesh] OR "Hospitals, Federal"[Mesh] OR "Hospitals, District"[Mesh] OR "Hospitals, County"[Mesh] OR "Hospitals, Convalescent"[Mesh] OR "Hospitals, Community"[Mesh] OR "Hospitals, Rehabilitation"[Mesh] OR "Hospice Care"[Mesh] OR "Hospices"[Mesh] OR "Hospice and Palliative Care Nursing"[Mesh])) OR ((swallow*[Title/Abstract] OR dysphag*[Title/Abstract] OR deglut*[Title/Abstract]) AND (Prevalence*[Title/Abstract] OR incidence*[Title/Abstract]) AND ((nursing[Title/Abstract] AND home[Title/Abstract]) OR (nursing[Title/Abstract] AND homes[Title/Abstract]) OR rehabilitation*[Title/Abstract] OR hospital[Title/Abstract] OR hospitals[Title/Abstract] OR hospice*[Title/Abstract] OR palliat*))	1294

### Methodological Quality Assessment

A quality appraisal of included studies was completed through consensus by the two independent reviewers, using the critical appraisal tool for cross-sectional studies, AXIS [22]. The AXIS appraisal tool is comprised of 20 questions that address common methodological issues and are arranged in an order that follow the general outline of a cross-sectional paper. Examples of issues addressed in the AXIS include clearly stated study aims, study design, sample size and selection, outcome variables measured, statistical analysis, non-response bias, reporting of results, justified discussion and conclusion, limitations, and ethics. AXIS questions that were answered “yes” were scored as “1” reflecting good methodological quality and, “no” was scored as “0” reflecting lower methodological quality. Two AXIS questions were formulated such that a positive answer “yes” would reflect negatively on methodological quality. Therefore, the scoring of these two questions was reversed in order to provide a uniform scoring method. The maximum total AXIS score possible was 20 being the best possible score for good methodological quality; however, not all items were applicable to every study. As such, total scores were converted into percentage scores: total score divided by the maximum score possible and multiplied by a hundred [23]. The level of evidence of the included studies was rated using the National Health and Medical Research Council (NHMRC) Evidence Hierarchy [24].

### Data Extraction

One reviewer extracted outcome data to an extraction table. Data were extracted regarding study setting and country, study population, definitions of terminology related to OD, OD screening and assessment methods, and OD prevalence data. A second reviewer performed a quality check of the extracted data. If necessary, authors of the included articles were contacted for clarification of terminology with regard to defining the setting [25–27] or to ask for access to raw data, when prevalence was described as a combination of different healthcare settings [28].

### Data Synthesis and Risk of Bias

Data extraction and study characteristics were retrieved using comprehensive data extraction forms. Assessment of the risk of bias was completed for each individual study using the AXIS critical appraisal tool [22]. Abstract selection, final study selection and quality assessments were the result of consensus-based ratings of two reviewers. Discrepancies were resolved through consensus with a third reviewer. Bias is not expected as the reviewers are not affiliated with any of the authors of the included studies.

### Meta-analysis

For the purpose of reducing heterogeneity for the meta-analysis and concerns regarding data completeness, quality, validity, reliability, and possible selection or recall bias, studies that collected prevalence data from notes in patient medical records, national databases, surveys, registries [29], or a dichotomous yes/no question to the patient or caregiver on the presence of a swallowing problem/difficulties [30] were not included in the meta-analysis. In addition, studies were excluded from the meta-analysis if it was not possible to compute proportional data results for screening or clinical assessment type and/or healthcare setting separately.

Data for subsampling were extracted from the included studies to measure the overall within- and between-group prevalence for different clinical settings: hospital, rehabilitation, and nursing home, according to the authors’ definition of the setting for each article. Overall within-group prevalence accounted for all studies with data for hospitals, rehabilitation, and nursing homes. Overall between-group prevalence was performed to determine confounding variables as a function of type of assessment method (e.g., screening, clinical non-instrumental assessment, instrumental assessment), diagnosis group, and type of hospital ward for each setting when applicable.

Meta-analysis of the prevalence of OD was completed using Comprehensive Meta-Analysis, Version 3.0 [31], providing estimates of pooled prevalence and forest plots. Due to the heterogeneity of the included studies, a random-effects model was used for summary statistics. Heterogeneity was estimated using the *Q* statistic to determine the spread of effect sizes about the mean and *I*^2^ to estimate the ratio of true variance to total variance. *I*^2^-values of less than 50%, 50% to 74%, and higher than 75% denote low, moderate, and high heterogeneity, respectively [32]. The classic fail-safe N test was used to assess publication bias. This test provides an estimate of the number of additional studies, with non-significant results, that would be necessary to add to the analysis in order to nullify the measured effect (N). A small N raises concern about the meta-analysis being compromised by publication bias; conversely, a large number suggests that it is unlikely that the meta-analysis is compromised by publication bias.

## Results

### Study Selection

**Fig. 1:**
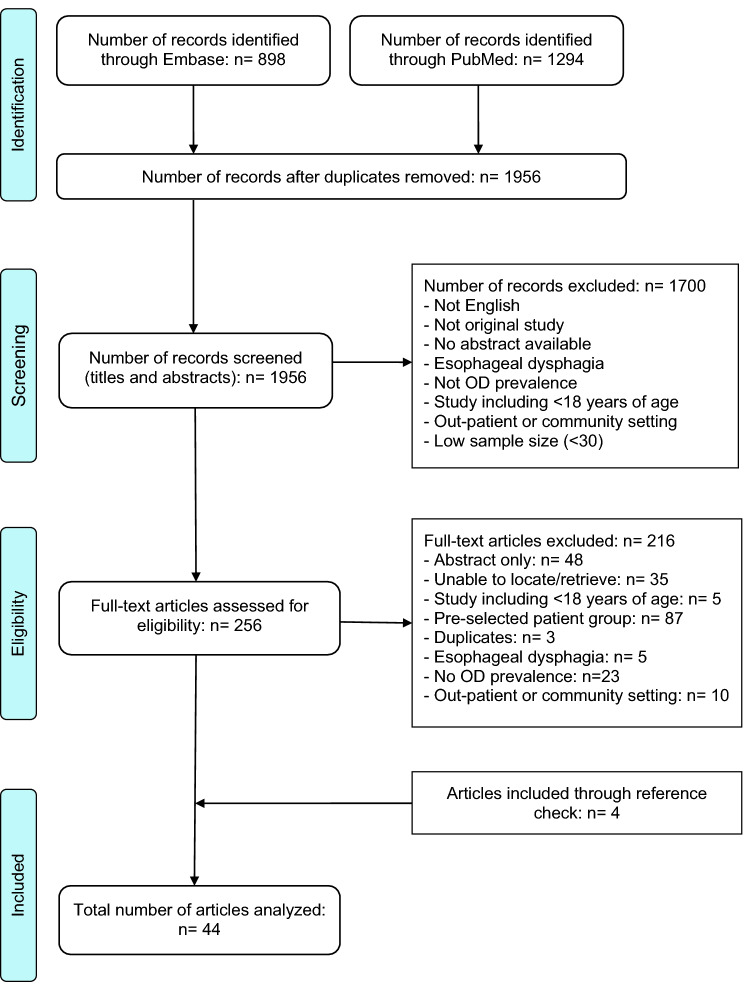
Flow diagram of the review process according to PRISMA [21]

The literature search and study selection results are illustrated in the PRISMA flow diagram (Fig. 1). The search resulted in 2192 records. After duplicates were removed, screening of the remaining 1956 records (abstracts and titles) resulted in 256 full-text articles assessed for eligibility. Forty articles were deemed eligible and an additional four articles were retrieved through reviewing of reference lists, resulting in inclusion of a total of forty-four articles.

### Synthesis of Methodological Quality

The methodological quality of the included studies was assessed by the AXIS critical appraisal tool. Higher AXIS scores indicate higher methodological quality of the studies being appraised, whereas lower scores identify methodological weaknesses that may result in poor reliability and validity of study results. The mean total score and percentages for all studies was 15.3 (SD 2.2; range 10–19) and 77% (SD 11; range 50–95), respectively. Two of the 44 studies scored 50% or lower [33, 34], 18/44 studies scored above 50% and below or equal to 75% [25–27, 35–49], and 24/44 studies scored above 75% [28, 50–72]. AXIS scores and percentages can be found in Table 2 and AXIS scores for each question in the Online Resource 1.

**Table 2:** Characteristics of studies included in the systematic review

Author (alphabetical order) Journal	Study design (NHMRC)^a^ AXIS score	Study setting and country Study period	Underlying medical diagnosis and recruitment criteria Time of screening/assessment	Sample characteristics: size, gender (male; female), age (years)	Description of OD terminology as used in study	Screening/Assessment for prevalence calculation Tester	Prevalence data
Abubakar et al. [35] Niger Postgrad Med J	Prospective cohort (II) AXIS 15/20; 75%	Tertiary teaching hospital, Nigeria April 2015–Jan 2017	Acute stroke *Inclusion:* consecutive inclusion at hospital (no specified ward; first acute stroke (ischemic and hemorrhagic); confirmed stroke with CT or MRI *Exclusion:* diseases that could interfere with swallowing: motor neuron disease, previous stroke, cerebral palsy, and chronic obstructive airway diseases; presented 1 week post-stroke onset (6); depressed sensorium (5) *Time of assessment:* within 72 h of first stroke	94 (*M* = 53; *F = *41) Mean age (SD): 55.5 (15.7)	*Dysphagia:* any difficulty associated with swallowing	- *3-oz water swallow* (DePippo et al.) [94] - *Swallow provocative test* (Warnecke et al.) [95] Dysphagia: - cough or wet/hoarse voice quality - latency of swallowing after either injection > 3 s. (Teramoto and Fukuchi) [96] Tester: NR	Overall Dysphagia 34.04% (32/94)* *Dysphagia per gender* *M* = 32.1% (17/53); *F = *36.6% (15/41) *Dysphagia per age group* < 65 years 32.8% (21/64); ≥ 65 years 36.7% (11/30) *NB Unclear prevalence calculation (i.e., fail one or both tests)*
Andrade et al. [50] Einstein (Sao Paulo)	Retrospective cross-sectional (IV) AXIS 16/20; 80%	Philanthropic hospital, Brazil Jan-Dec 2014	Mixed (gastrointestinal tract disease, cardiovascular disease, respiratory tract disorders, fractures, and neurologic diseases *Inclusion*: all adults and older individuals hospitalized, no specified ward *Exclusion:* intensive care unit, pregnant and puerperal women *Time of assessment:* NR	909 (*M* = 419; *F = *490) Mean age (SD): 54 (20.2) Age group: < 60: 534/909; ≥ 60: 375/909	*Dysphagia*: a swallowing difficulty in the passage of food from oral cavity to the stomach; at risk of dysphagia	- *Eating Assessment Tool EAT-10* Brazilian Portuguese version (Gonçalves et al.) [97] Dysphagia: EAT-10 score ≥ 3 Tester: NR	***Overall “risk” of dysphagia*** 10.5% (95/909) *Dysphagia per gender* *M* = 11.0% (46/419); *F = *10.0% (49/490) *Dysphagia per age group* < 60 years 6.9% (37/534); ≥ 60 years 15.5% (58/375)
Arnold et al. [51] PLoS One	Retrospective cohort (III-2) AXIS 18/19; 95%	Hospital, Switzerland Jan 2012-Nov 2013	Acute stroke *Inclusion:* consecutive acute ischemic stroke patients admitted to tertiary stroke center *Exclusion:* NR *Time of assessment:* within 24 h of admission	570 (*M* = 366; *F = *204) Mean age (SD): 65.1, (NR); Age range: (19.6–94.7)	*Dysphagia*	- *Data accessed from Bernese Stroke Registry:* - *Gugging Swallow Screen Test* *(GUSS)* (Trapl et al.) [98] Dysphagia: GUSS score of ≤ 19 GUSS score < 10 = severe dysphagia; required tube feeding Tester: physiotherapists experienced and trained in dysphagia	***Overall Dysphagia*** 20.7% (118/570) *Dysphagia per gender* *M* = 21.3% (78/366); *F = *19.6% (40/204) *Subgroup Dysphagia vs tube feeding (118/570)* without tube feeding*:* 68.6% (81/118); with tube feeding: 31.3% (37/118)
Baroni et al. [36] Arq Gastroenterol	Prospective cross-sectional (IV) AXIS 15/20; 75%	Tertiary hospital, Brazil May 2005-July 2006	Stroke *Inclusion:* consecutive patients admitted with (177) ischemic or (35) hemorrhagic cerebral vascular accident (CVA); clinical history of previous stroke *Exclusion:* any other neurological or structural changes that might interfere with swallowing process; inconclusive imaging exam (CT/MRI), coma and/or clinical ventilation with no possibility for clinical evaluation of swallowing *Time of Assessment:* 0–5 days of stroke (172/212); 11–20 days of stroke (14/212); 21–60 days of stroke (26/212)	212 (*M* = 125; *F = *87) Mean age (SD) 63.5 (NR) Age group: < 60 years (72/212); ≥ 60 years (140/212)	*Dysphagia, oropharyngeal dysfunction; swallowing dysfunction* *NB Use of terminology interchangeably throughout article*	- *Clinical evaluation:* a sample of liquid consistency (3, 5, 7 mL and/or a free volume of water), paste (3, 5, 7 mL and/or a free volume of thickened juice) & solids (free volume of cracker or bread) Swallowing dysfunction: one or more changes/alterations in any of the following: absence of lip seal, food escape, nasal reflux, residue in oral cavity, altered cervical auscultation, altered laryngeal elevation, vocal quality, resp. changes, cough, choking, fatigue, need for multiple swallows, compensatory maneuver during swallowing and escape of stained food through tracheostomy on blue dye test *NB Unclear if dysfunction must be present on all consistencies or only one*. *No cut off for cervical auscultation specified* Tester: speech therapist	***Overall Swallowing dysfunction*** 63% (134/212)* *Swallowing dysfunction per gender* *M* = 63.2% (79/125); *F = *63.2% (55/87) *Swallowing dysfunction per age group* < 60 years: 55.6% (40/72); 60 + years: 67.1% (94/140) *Subgroup swallowing dysfunction severity (134/212)* Mild 19% (26/134); Moderate 38% (51/134); Severe 43% (57/134)
Beharry, et al. [52] J Stroke Cerebrovasc Dis	Retrospective cohort (III-2) AXIS 16/20; 80%	Hospital stroke unit and/or intensive care, Switzerland January 1–December 31, 2015	Acute stroke *Inclusion:* all patients admitted within 24 h of acute ischemic stroke *Exclusion:* NR *Time of assessment:* Within 7 days of admission	340 (*M* = 183; *F = *157) Median age (IQR; range): 75 (18; 21–96)	*Swallowing disorders*	*- Data extracted from stroke registry;* patient medical records and notes of speech therapist and nurse - Modified version of Burke Dysphagia Screening Test (DePippo et al.) [99] Swallowing disorder if: 1. Cough or hoarse voice after screen = failed test OR 2. Patient complained of swallowing disorder without swallow screen and food texture had to be adapted OR 3. Patient required specialized speech therapy in first 7 days Tester: screening by nurse	***Overall swallowing disorders using combination of swallowing disorder criteria*** 23.6%(?) (81/340) ***Overall swallowing disorder- screening test*** 34.5% (51/148; n_missing_ = 192) *Swallowing disorder per gender* *M* = 49.4% (40/81) *F = *50.6% (41/81) *NB Inconsistencies in prevalence calculations within population tested*
Blanař et al. [28] J Adv Nurs	Retrospective cross-sectional (IV) AXIS 18/20; 90%	Multicenter; 237 departments in general hospitals geriatric hospitals, nursing homes and other healthcare facilities with ≥ 50 beds, Austria 2012–2016	Mixed (cancer, blood, digestive system, respiratory, psychological, cardiovascular, and musculoskeletal system diseases, dementia and diabetes mellitus) *Inclusion:* 18 years, available on day of measurement *Exclusion:* not available on day of measurement, refusal to participate, poor cognitive state *Time of assessment:* NR	17 580 (*M* = 7 489; *F = *10 091) Mean age (SD): 67.8 (18.1) Hospital Mean age (SD): 64.4 (NR) Nursing home Mean age (SD): 81.1 (NR)	*Dysphagia;* lower capacity to swallow, difficulty while swallowing, potentially unsafe while swallowing	*- Data extracted from database:* Dysphagia: ask the patient of she/he had problems swallowing Tester: 2 nurses	***Overall dysphagia all settings*** 6.6% (1155/17 580) ***Overall dysphagia hospital*** 5.3% (792/14 913) *Dysphagia per gender – hospital* *M* = 5.8% (398/6847) *F = *4.9 (394/8063) ***Overall dysphagia nursing home*** 13.6% (363/2667) Dysphagia per gender – nursing home *M* = 16.4% (105/642) *F = *12.7% (258/2025)
Brogan, et al. (1) [54] Dysphagia	Retrospective cohort (III-2) AXIS 17/20; 85%	Tertiary hospitals (6), Australia 2010	Stroke *Inclusion:* current admission of patients with primary diagnosis of stroke or CVA, regardless of stroke history; ischemic strokes 63.6% (339); hemorrhagic strokes 26.1% (139); not recorded 10.3% (55) *Exclusion:* subarachnoid or subdural hemorrhage; developed more than one infection (3) *Time of assessment:* on admission, days 3–4, and days 6–7	533 (*M* = 292; *F = *241) Mean age (SD): 71 (14.9) 378 follow-up after one week	*Dysphagia; swallowing problems*	*- Medical record review with standardized data collection form* Dysphagia: documented diagnosis by treating speech pathologist in medical record entry Tester: collected from medical records by speech pathologists *NB Specific criteria for dysphagia diagnosis was not specified*	***Overall Dysphagia*** 58.5% (?) (312/533) *NB Unclear where patient numbers come from (i.e., premorbid vs on admission?)* *Subgroup Dysphagia over time in follow-up group (378/533)* dysphagia on admission: 67.2%(?) (254/378); dysphagia on days 3–4: 52.1% (197/378); dysphagia on day 7: 46.0% (174/378) *NB Unclear inclusion criteria for follow-up group and reasons for decrease in dysphagia (i.e., resolved, deceased, *etc*.)* *Subgroup Nasogastric tube (NGT) in follow-up group 1st week* 18.4% (98/532; n_missing_ = 1); NGT within 1st week; had dysphagia on admission 95.9%(?) (93/98)
Brogan et al. (2) [53] Neuroepidemiology	Retrospective cohort (III-2) AXIS 17/20; 85%	Tertiary hospitals (6), Australia 2010	Stroke *Inclusion:* current admission of patients with primary diagnosis of stroke or CVA, regardless of stroke history *Exclusion:* subarachnoid or subdural hemorrhage; developed more than one infection (3) *Time of assessment:* first week post-stroke	533 (*M* = 292; *F = *241) Mean age (SD): 71 (14.9)	*Dysphagia; swallowing problems*	- *Medical record review with standardized data collection form* Dysphagia: based on documented diagnosis by treating speech pathologist in medical record entry Tester: speech pathologist	***Overall Dysphagia*** 61.5% (328/533) *NB Inconsistencies in recorded dysphagia prevalence from same study sample as Brogan *et al*.* [53]* (1)*
Carrión et al. [55] Clin Nutr	Prospective cohort (II) AXIS 18/19; 95%	Hospital, acute geriatric unit, Spain Jan 1 2005-Dec 31 2009	Mixed acute illness (diseases of the circulatory, respiratory, genitourinary, and digestive systems) *Inclusion:* consecutive, ≥ 70 years; acute disease; able to undergo V-VST *Exclusion:* none *Time of assessment*: upon admission	1662 (*M* = 637; *F = *1025) Mean age (SD): 85.1 (6.23)	*Oropharyngeal dysphagia (OD*)	*- Volume-viscosity swallow test* *(V-VST)* (Clavé et al.) [74] Oropharyngeal dysphagia: impairment in efficacy and/or safety of the swallow Tester: geriatric unit nurse trained in V-VST; speech pathologist support when in doubt	***Overall Oropharyngeal dysphagia*** 47.4% with 95% CI 45–49.8 (788/1662)* *Oropharyngeal dysphagia per gender* *M* = 47.6% (303/637); *F = *47.3% (485/1025)
Chen et al. [37] BMC Geriatrics	Prospective cross-sectional (IV) AXIS 13/20; 65%	Nursing homes (18), China Study period: May–July 2019	NR *Inclusion:* ≥ 60 years; ability to answer questions or have help from caregivers familiar to situation; written consent from participant or family member *Exclusion:* intellectual disabilities *Time of assessment:* NR	775 (*M* = 305; *F = *470) Mean age (SD): 81.3 (9.5) Age group: 60–69 years (93/775) 70–79 years (192/775) ≥ 80 years (490/775)	*Dysphagia; symptoms and signs of dysphagia*	*- Eating Assessment Tool (EAT-10)* (Belafsky et al.) [75] *NB no reference for Chinese version of EAT-10* *Risk of dysphagia: total EAT-10 score* ≥ *3* Tester: trained nurses and post-graduate students	***Overall “risk” of dysphagia*** 31.1% (241/775)*** *Risk of dysphagia per gender* *M* = 41.9% (101/241); *F = *58.1% (140/241)
Crary et al. [56] Dysphagia	Prospective cohort (II) AXIS 16/20; 80%	Tertiary hospital, primary stroke center, USA Study period: NR	Acute stroke *Inclusion:* consecutive, acute ischemic stroke *Exclusion:* pre-stroke history OD, head/neck surgery or trauma, neurological disorder to impact swallowing ability *Time of assessment:* on admission	67 (*M* = 29; *F = *38) Mean age (SD): 65.7 (NR)	*Dysphagia*	*- Mann Assessment of Swallowing Ability* *(MASA)* (Mann) [76] Tester: speech-language pathologist trained in MASA	***Overall dysphagia*** 37.3% (25/67)* *Oropharyngeal dysphagia per gender* *M* = 34.5% (10/29); *F = *39.5% (15/38)
De Cock et al. [57] Eur J Neurol	Prospective cross-sectional (IV) AXIS 19/20; 95%	University hospital, stroke unit, Belgium March 2018-October 2019	Stroke *Inclusion:* diagnosis first-ever ischemic stroke; ≥ 18 years, Dutch speaking, admitted within 48 h after onset of acute stroke symptoms *Exclusion:* history of other diseases influencing swallowing, speech and or language (e.g., dementia, Parkinson’s disease, oral carcinomas, mental retardation) *Time of assessment:* immediately upon arrival at stroke unit	151 (*M* = 85; *F = *66) Mean age (SD; range): 67 (14; 25–79)	*Dysphagia*	*- Standardized water-swallowing test* (90 ml) (De Bodt et al.) [100] Dysphagia: suspected penetration or aspiration of liquids Failed screening: referred to bedside examination with Mann Assessment of Swallowing Ability (MASA) to discard or confirm dysphagia; some of these patients referred to FEES or VFSS Functional Oral Intake Scale (FOIS) was taken on dysphagic stroke patients Tester: trained speech-language pathologist	***Overall dysphagia*** 23% (35/151)* *Dysphagia per gender* *M* = 22.4% (19/85); *F = *24.2% (16/66) *NB no reported data for VFSS, FEES or FOIS*
Diendéré et al. [38] Nutrition	Prospective cohort (II) AXIS 14/20; 70%	Hospitals (2), Burkina Faso November 2015 – August 2016	Stroke *Inclusion:* stroke: 59.9% (133) ischemic; 40.1% (89) hemorrhagic *Exclusion:* comatose, early discharge, no consent (336) *Time of assessment:* admission (Day 0), day 8 and day 14 (if clinical notification of dysphagia had been made of dysphagia between day 8 and day 14) Admission was 2.3 (1.4) days after stroke	222 (*M* = 121; *F = *101) Mean age (SD): 60.5 (14.2) Age group < 65 years (130/222) ≥ 65 years (92/222)	*Dysphagia*	- *Practical Aspiration Screening Schema system* (Zhou et al.) [101]: a combination of Echelle Clinique Prédictive de Fausse Route (Guinvarch et al.) [102] and DePippo 3 oz test (DePippo et al.) [94] Dysphagia: < 14 points on Echelle Clinique Predictive de Fausse Route or; 14–28 points on Echelle Clinique Predictive de Fausse Route and producing cough or wet or gargly voice within a minute after 3 oz water test Tester: students of medicine	***Overall Dysphagia*** day 0: 37.4% (95% CI: 31.0–44.1) (83/222)* *Subgroup dysphagia* day 8: 28.4% (95% CI: 22.2–35.3) (56/197); day 14*:* 15.8% (95% CI: 10.8–21.8) (29/184)
Falsetti et al. [58] J Stroke Cerebrovasc Dis	Prospective cohort (II) AXIS 16/18; 89%	Hospital; Neurorehabilitation unit, Italy January 2005- December 2006	Stroke *Inclusion:* consecutive patients admitted with previous ischemic 74.1% (112) and hemorrhagic 25.8% (39) stroke *Exclusion:* patients with history of head and neck damage, neurologic disease other than cerebrovascular disorder, previous dysphagia Mean duration of disease (time from stroke) 14 days *Time of assessment:* clinical bedside test within 24 h of admission; videofluoroscopy (VFSS) within first week of admission	151 (*M* = 77; *F = *74) Mean age (SD): 79.4 (6.2)	*Dysphagia: disorder of deglutition affecting the oral, pharyngeal and/or esophageal phases of swallowing; oropharyngeal dysphagia*	- *Clinical bedside test:* Step 1) identify level of consciousness and collaboration (patients with level of cognitive functioning (LCF) < 4 were immediately considered dysphagic) and oral motor and sensory assessment (voice quality; speech and language; swallowing of saliva; movements of cricoid cartilage; lips, tongue, and velopharynx; gag reflex; preservation of pharyngeal sensation; capability of voluntary cough) Step 2) swallowing of 5 mL of water with concomitant pulse oximetry, observing signs of oral-facial apraxia (loosening of water from lips, delay in swallowing, abnormality or absence of tongue movements) or signs of penetration /aspiration (‘‘wet’’ or ‘‘gurgly’’ voice, coughing, > 2% decrease of basal value of oxygen saturation at pulse oximetry Step 3) swallowing at least 20 mL of water, with the same procedures above Dysphagia: abnormality in ≥ 1 item in any of the above steps - Videofluoroscopy (VFSS) with standard protocol (on patients who failed clinical testing within first week of admission) -patient swallowed in sequence 5 ml and 10 ml of solution of barium of different consistency (liquid at later attempts of the examination) Cessation of the VFSS if significant aspiration Dysphagia: Classified as no dysphagia; dysphagia affecting oral phase; dysphagia affecting pharyngeal phase; mixed dysphagia Ordinal penetration–aspiration scale was used for scoring airway invasion Tester: doctor	***Overall Dysphagia (clinical bedside test)*** 41% (62/151)** ***Overall Dysphagia per phase (clinical bedside test)**** (62/151)* prevalently oral phase 15.2% (23/151); prevalently pharyngeal *phase* 9.2% (14/151); prevalently mixed dysphagia 16.6% (25/151) *Dysphagia per gender (clinical bedside test)* *M* = 40.3% (31/77); *F = *41.9% (31/74) ***Overall Dysphagia (VFSS)* (49/151)** 93.9% (46/49) ***Overall Dysphagia per phase (VFSS)*** prevalently oral phase 9.9% (15/151); prevalently pharyngeal phase 8.6% (13/151); prevalently mixed dysphagia 9.9% (15/151) ***Overall Penetration/aspiration (VFSS)*** penetration: 13.9% (21/151); aspiration: 8.6% (13/151); silent and significant aspiration: 4% (6/151) ***Overall dysphagia with PEG tube*** 11.9% (18/151)
Finestone et al. [59] Arch Phys Med Rehabil	Prospective cross-sectional (IV) AXIS 17/20; 85%	Tertiary hospital; Rehabilitation unit, Canada 14 months	Stroke *Inclusion:* consecutively admitted stroke patients from acute care at same hospital *Exclusion:* patients declined to participate (4) *Time of assessment:* within 4 days of admission, 1 & 2 months, follow-up 2–4 months post-discharge	49 (*M* = 32; *F = *17) Mean age (SD): *M* = 60 (NR); *F = *62 (NR) Age range: *M* = 20–77; *F = *20–78	*Dysphagia*	- *Clinical observation* Dysphagia: observed choking, coughing, exhibiting vocal quality changes (i.e., wet-sounding voice after food consumption), decreased oral motor function (i.e., weakness on right and/or left sides) or having difficulty swallowing Dysphagia was then verified by bedside assessment or an abnormal videofluoroscopic modified barium swallow Tester: swallowing team; radiologist and/or speech-language pathologist, dietitian & occupational therapist	***Overall Dysphagia*** 47% (23/49)** ***Overall Tube feedings*** 14% (7/49) ***Overall dysphagia 1 month*** (32/49) 34% (11/32); *Dysphagia 2 months (9/32)* *33% (3/9)*
Flowers et al. [60] J Commun Disord	Retrospective cohort (III-2) AXIS 16/20; 80%	Tertiary hospital, Canada July 1, 2003–March 31, 2008	Stroke *Inclusion:* select patients within 2 weeks of first acute stroke or transient ischemic attack (TIA); ≥ 18 years; had diffusion weighted MR imaging (median time of imaging from stroke onset 75 h /IQR 108 h) *Exclusion:* random selection of 250 (466); irretrievable data (29) *Time of assessment:* median of 2 days /IQR 3 days after stroke onset	221 (*M* = 124; *F = *97) Mean age (SD): 68 (15)	*Dysphagia: oropharyngeal dysphagia characterized by abnormal swallowing physiology of the upper aerodigestive tract*	*- Registry of Canadian Stroke Network’s database; Medical chart review* - document the number of patients assessed by speech-language pathologists (SLP) Dysphagia: identified by SLP clinical or instrumental assessment, or presence of enteral feeding tube (NGT) insertion in patients not assessed by SLP Tester: trained research assistant extracted data	***Overall Dysphagia*** (identified by SLP or NGT insertion) 44% (98/221) (95% CI 38–51) ***Overall Dysphagia* (identified by SLP*)*** 40.7% (90/221) ***Overall Dysphagia* (identified by NGT insertion)** 3.6% (8/221) *Dysphagia per gender* *M* = 46.8% (58/124); *F = *41.2% (40/97)
Gordon et al. [39] Br Med J (Clin Res Ed)	Prospective cohort (II) AXIS 15/20; 75%	District general hospital, England 6 months	Acute stroke *Inclusion:* consecutive clinical diagnosed acute stroke *Exclusion:* stroke > 14 days prior to admission or not stroke diagnosis (9) *Time of assessment:* ≤ 2 days (56/91); ≤ 3 days (26/91); ≤ 13 days (9/91) after stroke	91 (*M* = 38; *F = *53) Median age (Q_1_; Q_2_): 70 (NR) Age range: 26–96	*Dysphagia*	*- Modified Frenchay dysarthria assessment* (Enderby 1983) [103] Dysphagia: inability to drink 50 ml water or choking more than once while attempting to drink 50 ml on 2 occasions Tester: neurologist	***Overall Dysphagia*** 45.1% (41/91)* *Dysphagia per gender* *M* = 47.4% (18/38); *F = *43.4% (23/53)
Groher and Bukatman [40] Dysphagia	Prospective cross-sectional (IV) AXIS 12/18; 67%	Hospitals (2); primary, secondary and tertiary care, USA April 1985	Mixed (CVA, central nervous system/dementia, head and neck cancer, trauma, neurodegenerative diseases, Guillain-Barré, Multiple Sclerosis, Parkinson’s disease, Huntington’s disease, ALS, gastrointestinal and middle-stage systemic diseases) *Inclusion:* various disorders; only patients identified by both examiners *Exclusion:* pre-operative or post-operative course with expected swallowing dysfunction as a result; hospital services including psychiatry, pediatrics, neonatology, obstetrics, and inpatient substance abuse; not identified by both examiners (2) *Time of assessment:* NR	1072 Hospital 1 = 462 Hospital 2 = 610 (*M* = NR; *F = *NR) Mean age (SD): NR	*Swallowing dysfunction:* oral ingestion or pharyngeal motility disorders; swallowing disorders	*- Cardex review:* -standard form; missing information was gathered from medical records Swallowing dysfunction: presence of one of two criteria: -reported signs of choking, drooling or inability to complete an attempted swallow; -history of aspiration pneumonia with a diagnosis of primary or secondary neuromuscular disease Tester: hospital service’s head nurse and investigator from each hospital	***Overall Swallowing dysfunction*** 12.2% (131/1072) Hospital 1: 12% (54/462) Hospital 2: 13% (77/610) *NB Inconsistencies of numbers in table and reported results*
Hollaar et al. [72] Geriatr Nurs	Retrospective cross-sectional (IV) AXIS 17/20; 85%	Nursing homes (3), Netherlands Nursing home 1: April 2011-April 2012 Nursing home 2: April 2012-April 2013 Nursing home 3: April 2013-April 2014	NR *Inclusion:* ≥ 65 years; variety of diagnosis *Exclusion:* residents discharged during examination period (43) *Time of assessment:* NR	373 (*M* = 113; *F = *260); Mean age (SD): 83.3 (8.0) Nursing home 1: (*M* = 30; *F = *50); Mean age (SD): 79.2 (7.6) Nursing home 2: (*M* = 20; *F = *66); Mean age (SD): 85.2 (5.4) Nursing home 3: (*M* = 63; *F = *144); Mean age (SD): 84.2 (8.6)	*Dysphagia*	*- Medical electronic file review* Dysphagia if any of the following: -history of nursing home-acquired pneumonia (NHAP); -report of clinical swallowing assessment by speech therapist at nursing home; -confirmation by consulting speech therapist or elderly care physician (in case of doubt) Tester: researchers	***Overall Dysphagia*** *16%* (59/373)
Huppertz et al. [41] J Nutr Health Aging	Retrospective cross-sectional (IV) AXIS 15/20; 75%	Nursing home, Netherland 1 day in 2016 or 2017	NR *Inclusion:* ≥ 65 years; living in somatic- and psychogeriatric wards; include only 2017 data if resident participated in both years *Exclusion:* residents that received palliative care at the day of the measurements *Time of assessment:* NR	6349 (*M* = 1892; *F = *4457) Mean age (SD): 84.5 (7.5) *Psychogeriatric wards*: 66% (4190/6349); *Somatic wards:* 34% (2159/6349)	*Oropharyngeal dysphagia: swallowing problems*	*- Standardized questionnaire: National prevalence measure of quality of care which included 2 questions related to oropharyngeal dysphagia:* 1) “Does the client have swallowing problems?” 2) “Does the client sneeze or cough while swallowing food or liquids?” Swallowing problems: answer yes to question(s) *NB Unclear if one or both questions need to be answered “yes” to be classified swallowing problems* Tester: trained nurses	***Overall swallowing problems*** 12.1% (769/6349) ***Overall Sneeze/cough while swallowing*** *6.9% (439/6349)* ***Overall Swallowing problems with additional sneeze/cough while swallowing*** 5.6% (361/6349) *Swallowing problems per gender* *M* = 40.6% (769/1892); *F = *12% (499/4457)
Hägglund et al. [25] Dysphagia	Prospective cross-sectional (IV) AXIS 15/20; 75%	Short-term care units; five counties, Sweden Study period: NR	NR *Inclusion:* ≥ 65 years old; diagnosis causing short- term care *Exclusion:* declined to participate (63) *Time of assessment:* NR	391 (*M* = 182; *F = *209) Median age (Q_1_;Q_2_): 84 (NR) *M* = 81 (NR) *F = *85 (NR) Age years: < 75 16.1% (63/391) 75–84 38.1 (149/391) ≥ 85 45.8% (179/391) Age range: *M* = 65–98; *F = *65–110	*Swallowing dysfunction; dysphagia* Swallowing capacity: volume of swallowed water divided by time	*- Teaspoon test with 3 teaspoons of water* (prior to WST): if aspiration signs, no WST (score: 0 mL/s) *- Timed water swallow test* *(WST)* (Nathadwarawala et al.) [104] Swallowing dysfunction: abnormal swallowing capacity and/or signs of aspiration Abnormal swallowing capacity: unable to swallow > 10 mL/s Signs of aspiration: cough, voice change, and/or voice change without cough after swallow Tester: eight registered dental hygienists and one speech-language pathologist with specific training on all assessments	***Overall Swallowing dysfunction*** *63.4%* (248/385; n_missing_ = 6)*** ***Overall Abnormal swallowing capacity*** 55% (213/385; n_missing_ = 6) ***Overall Signs of aspiration*** 34% (127/377; n_missing_ = 14); ***Overall cough*** 24% (90/377; n_missing_ = 14); ***Overall voice change*** 18% (65/368; n_missing_ = 24); ***Overall voice change without cough*** 10% (37/368; n_missing_ = 24) *Swallowing dysfunction per gender* *M* = 64% (116/180; n_missing_ = 2); *F = *64% (132/205; n_missing_ = 4) *Abnormal swallowing capacity per gender* *M* = 49% (89/180; n_missing_ = 2); *F = *60% (124/205; n_missing_ = 4) *Swallowing dysfunction per age group* < 75 12.9% (32/62); 75–84 37.5% (93/148); ≥ 85 49.6% (123/175) *Abnormal swallowing capacity per age group* < 75 13.6% (29/62); 75–84 36.6% (78/148); > 85 49.8% (106/175) *Signs of aspiration per gender* *M* = 39.1% (70/179; n_missing_ = 3); cough 28.5% (51/179; n_missing_ = 3); voice change 22%% (39/177; n_missing_ = 5); voice change without cough 10% (37/177; n_missing_ = 5); *F = *28.8% (57/198; n_missing_ = 11); cough 19.7% (39/198; n_missing_ = 11); voice change 13.6% (26/191; n_missing_ = 18); voice change without cough 9.4% (18/191; n_missing_ = 18)
Jørgensen et al. [61] Clin Nutr ESPEN	Prospective cross-sectional (IV) AXIS 16/19; 84%	Hospitals (3), Denmark June –October 2016	Mixed (infection/fever, dehydration/dizziness/fall, pneumonia/aspiration pneumonia, pulmonary disease, poor general condition/diarrhea, other) *Inclusion:* consecutive patients admitted to medical or geriatric wards *Exclusion:* if previous contact with occupational therapist during admittance; not sufficiently alert to give informed consent and participate in test; language barrier for informed consent and test participation *Time of assessment:* NR	110 (*M* = 46; *F = *62) Mean age (SD): 75 (12.4)	*Oropharyngeal dysphagia (OD*)	*- Volume-Viscosity Swallow Test (V-VST)* (Clavé et al. 2008) [74] Oropharyngeal dysphagia: one or several signs of impaired safety and/or efficacy during trials the V-VST Signs of impaired safety: cough, changes in voice quality, a decrease in oxygen saturation ≥ 3% Impaired efficacy: impaired labial seal, oral residue, piecemeal deglutition, pharyngeal residue Testers: occupational therapists, trained in V-VST	***Overall Oropharyngeal dysphagia*** 34.5% (38/110)* ***Overall signs of impaired safety*** 6.4% (7/110); ***Overall signs of impaired efficacy*** 16.4% (18/110); ***Overall signs of both impaired safety and efficacy*** 11.8% (13/110)
Kampman et al. [62] Neurohospitalist	Retrospective (period I) & Prospective (period II) cross-sectional (IV) AXIS 14/18; 78%	University hospital, Norway Period I; June 1, 2012–May 31 2013 Period II; December 1, 2013–May 31, 2014	Stroke *Inclusion:* patients admitted with cerebral infarction or intracerebral hemorrhage on day of stroke or the day after; stayed in stroke unit for at least 48 h after admission *Exclusion:* patients receiving terminal care only; those who died during the first 2 weeks after the stroke *Time of assessment:* 0–1 days	Period I 199 (*M* = 110; *F = *89) Median age (Q_1_;Q_2_): 75 (NR) Age range: 20–94 Period II 86 (*M* = 53; *F = *33) Median age (Q_1_;Q_2_): 75 (NR) Age range: 22–92	*Dysphagia; swallowing problems*	*- Swallow test* *1 teaspoons water 3 times;* if cough, give teaspoon thick liquid; if cough again, stop test and contact speech therapist or other qualified personnel; if swallowing is okay, have patient drink 1/3 glass of water (about 50 ml) with or without thickener *NB Unclear criteria for dysphagia (i.e., cough on one or all trials)* Tester: NR	***Overall Dysphagia*** 23.2% (57/285n_missing_ = 40)* Period I: 23.3%(?) (39/168n_misssing_ = 31) Period II: 23.4% (18/77n_missing_ = 9) *NB overall prevalence reported for total included, not tested*
Kidd et al. [42] Q J Med	Prospective cohort (II) AXIS 14/20; 70%	Hospital, UK Study period: NR	Acute stroke *Inclusion:* consecutive, first acute stroke; conscious; *Excluded:* other neurological disorder that may give rise to dysphagia or dysphagia due to other reasons; unable to obtain verbal consent from the patient or their next of kin *Time of assessment:* Within 72 h of stroke onset; Re-assessment Day7 and Day14 after stroke and 3 months	60 (*M* = 25; *F = *35) Mean age (SD): 72 (9.5)	*Dysphagia; aspiration; swallowing problem*	*- Water-swallowing screen and videofluoroscopy* (VFSS) Screen: 50 ml water swallow test given in 5 ml aliquots Dysphagia: cough, choke, altered voice quality, fail water swallow test Videofluoroscopy determined aspiration *NB not all patients with dysphagia aspirated and some patients who aspirated did not test positive for dysphagia* Tester: NR	***Overall dysphagia water swallow *** 42% (25/60)* *Subgroup dysphagia—over time* Day 7 19% (10/51); Day 14 10% (4/37)
Lindroos et al. [27] J Nutr Health Aging	Prospective cross-sectional (IV) AXIS 15/20; 75%	Assisted living facilities (33), Finland 2007	Mixed (Dementia, stroke, Parkinson’s disease, COPD, chronic or recurrent infections) *Inclusion:* residents ≥ 65 years *Exclusion:* refused (628); admitted for temporary respite care (111); did not have swallowing data available (9) *Time of assessment*: NR	1466 (*M* = 323; *F = *1143) Mean age (SD): 83 (NR)	*Swallowing difficulties; dysphagia:* a difficulty or discomfort during progression of a bolus from the mouth to the stomach	*- Structured questionnaire with patient and closest caregiver* Swallowing difficulties: - answered yes to question asking if they experienced swallowing difficulties; - had observed difficulties at bedside assessment; or had prior difficulties observed with resident’s eating and feeding Tester: trained nurses	***Overall Swallowing difficulties*** 11.8% (173/1466) *Swallowing difficulties per gender* *M* = 15% (26/323); *F = *85% (147/1143)
Mañas-Martinez et al. [43] Endocrinol Diabetes Nutr	Retrospective cohort (III-2) AXIS 12/20; 60%	Tertiary Hospital, Spain January-March 2012 Follow-up via electronic case history until April 2014	Mixed (pneumonia, heart failure, anemia, urinary tract infection, other) *Inclusion:* acute disease admitted to internal medicine ward *Exclusion:* unable to cooperate in the EAT-10 test due to advanced dementia or severe neurological disease in the absence of a proxy *Time of assessment:* within 48 h of admission	90 (*M* = 56; *F = *34) Mean age (SD): 83 (11.8)	*Oropharyngeal dysphagia (OD*); at risk of OD	*- Medical chart review* - *Eating Assessment Tool (EAT-10)* (Belafsky et al.; Burgos et al.) [75, 105] OD: EAT-10 score of ≥ 3 Tester: NR	***Overall OD*** 56.7% (51/90)
Mateos-Nozal et al. [63] JAMDA	Prospective cross-sectional (IV) AXIS 18/20; 90%	University hospital, acute geriatric unit, Spain Study period: NR	Mixed acute (heart failure, respiratory infection, urinary tract infection, abdominal infection, other) *Inclusion:* ≥ 80 years *Exclusion:* previously included in the study (84), end of life situation (68); permanent low level of consciousness (43); no consent (12); enteral nutrition (10); not tested within first 2 days of admission (31) *Time of assessment:* ≤ 48 h of admission	329 (*M* = 104; *F = *225) Mean age (SD); 93.5 (4.1)	*Oropharyngeal dysphagia (OD);* Difficulty forming or moving bolus from oral cavity to esophagus	*- Volume-Viscosity Swallow Test- (V-VST)* (Clavé et al.) [74] OD: one or several signs of impaired safety and/or efficacy during trials the V-VST Signs of impaired safety or efficacy: cough, changes in voice quality, a decrease in oxygen saturation, poor labial seal, multiple swallows, and oropharyngeal residue Tester: trained nurse	***Overall OD*** 82.4% (271/329)* *OD per gender* *M* = 76.9% (80/104); *F = *84.9% (191/225)
Melgaard, Rodrigo-Domingo and Mørch [64] Geriatrics	Prospective cross-sectional (IV) AXIS 18/20; 90%	Regional hospital, Denmark March 1–August 31, 2016	NR *Inclusion:* consecutively admitted to geriatric medicine department; ≥ 60 years old, hospitalized minimum 24 h; able to cooperate in OD test *Exclusion:* not tested for OD; did not meet inclusion criteria; did not want to participate *Time of assessment:* NR	313 (*M* = 156; *F = *157) Mean age (SD): 83.1 (7.81)	*Oropharyngeal dysphagia (OD):* difficulties moving food from the mouth to stomach	*- Volume-Viscosity Swallow Test* *(V-VST)* (Clavé et al.) [74] *- Minimal Eating Observation Form version II (MEOF-II*) (Westergren et al.) [106] Oropharyngeal dysphagia: -one or more signs of impaired safety or efficacy: changes of voice quality, cough, or decrease in oxygen saturation ≥ 3% on V-VST; -a dysfunction in ingestion or deglutition on MEOF-II Tester: trained and experienced occupational therapists	***Overall Oropharyngeal dysphagia*** 50% (156/313) *Oropharyngeal dysphagia per gender* *M* = 46.2% (72/156); *F = *53.5% (84/157) *NB Unclear prevalence calculation (i.e., fail one or both tests)*
Nielsen et al. [65] Clin Nutr ESPEN	Prospective cross-sectional (IV) AXIS 17/20; 85%	Regional hospital, Denmark March 1, 2016–September 1, 2016	Mixed (cardiopulmonary problems, osteoarticular disease, dementia and psychiatric diseases) *Inclusion:* admitted to acute geriatric department; ≥ 65 years old; hospitalized minimum 24 h *Exclusion:* terminal stage illness, severe dementia, in some other way unable to participate, did not wish to participate or transferred from department (105); lack of or insufficient screening (16) *Time of assessment:* NR	297 (*M* = 130; *F = *167) Mean age (SD): 83 (7.7)	*Eating difficulties; deglutition; swallowing difficulties*	*- MEOF-II screening instrument* (Westergren et al.) [106] Eating difficulties: includes 3 components: ingestion, deglutition and energy/appetite; Dichotomous rating: yes/no Deglutition defined: Problems in manipulation of food in mouth, swallowing difficulties and difficulties in chewing Tester: occupational therapists trained in screening	***Overall eating difficulties*** 55% (163/297) **Ingestion** difficulties in sitting position 13.4% (40/297); difficulties in manipulation of food on the plate 23.2% (69/297); difficulties in transport of food to the mouth 20.9% (62/297) **Deglutition** problems in manipulation of food in the mouth 26.6% (79/297); swallowing difficulties 28% (83/297); difficulties in chewing 27.6% (82/297) **Energy/appetite** eats less than ¾ of served portion 15.2% (45/297) ***Overall Eating difficulties per gender*** *M* = 53% (69/130); *F = *56.3% (94/167) *NB overall OD includes data not related to OD*
Nogueira and Reis [44] Clin Interv Aging	Prospective, cross-sectional (IV) AXIS 11/20; 55%	Nursing homes (8), Portugal Study period: NR	NR *Inclusion:* all nursing home residents *Exclusion:* did not sign informed consent (6) *Time of assessment:* NR	266 (*M* = 66; *F = *200) Mean age (SD): 82 (10)	*Swallowing disorders; deglutition: may involve both oral and pharyngeal stage of swallowing; swallowing and eating impairments*	*- 3 oz Water Swallow Test* *(3 oz WST)* (DePippo et al.) [94] Swallowing disorders: stop drinking, cough, choke, or have a wet-hoarse vocal quality during the test or for 1 min afterward *- The Dysphagia Self-Test* *(DST)* questionnaire was administered to the residents who were able to respond (Logemann et al.) [107] Signs of dysphagia: ≥ 7 difficulties Tester: trained researchers, speech-language therapist	***Overall Swallowing disorders with 3 oz WST*** 38.2%(?) (64/234?) ***Overall signs of dysphagia on DST (*≥ *7 difficulties)*** 40.1% (90/224; n_missing_ = 42) *NB Unclear prevalence calculation within reported population tested on WST*
Paciaroni et al. [66] Eur Neurol	Prospective cross-sectional (IV) AXIS 14/18; 78%	University hospital, Italy April 2001 – December 2002	Acute stroke *Inclusion:* consecutive, acute first stroke (ischemic 343; hemorrhagic 63) admitted to stroke unit; conscious (GCS ≥ 10); medically stable *Exclusion:* patients with a history of previous swallowing impairment or medical condition that could affect swallowing function *Time of assessment:* immediately after hospitalization and every 24 h until recovery from dysphagia Mean time from stroke and first assessment 330 min (range 60–720 min)	406 (*M* = 219; *F = *187) Mean age (SD): 73.2 (11.4)	*Dysphagia; swallowing impairment; swallowing dysfunction*	*- Clinical bedside assessment* (Mann et al.; Warlow et al.) [92, 108] Dysphagia: if possible, probable or definite from clinical assessment (Mann et al.) [92] Tester: neurologist	***Overall dysphagia *** 34.7% (141/406)* *Dysphagia per gender* *M* = 31% (68/219); *F = *39% (73/187) *Subgroup Dysphagia per stroke type* ischemic stroke 32.1% (110/343); hemorrhagic stroke 49.2% (31/63)
Park et al. [67] Geriatr Nurs	Prospective cross-sectional (IV) AXIS 16/20; 80%	Nursing homes (2), South Korea July – August 2010	NR *Inclusion*: nursing homes with: more than 5 years operation; more than 115 staff members; more than 100; resident’s ≥ 65 years *Exclusion:* 6 of 8 nursing homes meeting inclusion criteria did not wish to participate; residents unable to follow instructions (47); resident refusal or unable to acquire consent (40) *Time of assessment:* NR	395 (*M* = 93; *F = *302) Mean age (SD): 80.7 (8.0) Age group: < 74 years: 92 ≥ 75 years: 303	*Dysphagia: swallowing impairment*	*- Korean version of Gugging Swallowing Screen Test* *(GUSS)* (Lee et al.; Trapl et al.) [98, 109] Dysphagia: GUSS score 0–14 Severity of risk of aspiration: GUSS score 0 to 9: high; GUSS score 10–14: moderate; GUSS score 15–19: low; GUSS score 20: minimal Tester: five research assistants (experienced and trained registered nurses)	***Overall dysphagia*** 52.7% (208/395)*** ***Overall Aspiration risk*** High risk of aspiration 41.1% (162/395); Moderate risk of aspiration *11.6*% (46/395) *Dysphagia per gender* *M* = 61.3% (57/93); *F = *50% (151/302) *Dysphagia per age* < 74 years 43.5% (40/92); ≥ 75 years 55.4% (168/303)
Patel and Martin [45] J Nutr Health Aging	Prospective cross-sectional (IV) AXIS 14/20; 70%	Hospital, UK May 1999- February 2000	Mixed acute illness (chest infection, acute exacerbation of chronic lung diseases, pulmonary edema, gastro-enteritis, and gastrointestinal bleeding) *Inclusion:* patients admitted to elderly care unit within preceding twenty-four hours of 9am on Mondays and Thursdays *Exclusion:* NR *Time of assessment:* After admission preceding 24 h of 9 am Monday and Thursdays	100 (*M* = 27; *F = *73) Mean age (SD): 81.7 (NR) Age range: 65–98	*Dysphagia*	*- Nurse clinical observations, food-charts, case-notes, unstructured interviews of patients and*/*or carers* -estimate for whether subject had adequate intake; consumed at least ¾ of their standard diet and any prescribed food supplements -categorization of the reasons for inadequate intake: acute illness, anorexia, oral problems, low mood confusion, catering limitations and dysphagia Dysphagia: NR *NB Unclear criteria for determining dysphagia* Tester: researchers	***Overall dysphagia *** 6% (6/100)
Rofes et al. [68] Neurogastroenterol Motil	Prospective cohort (II) AXIS 18/20; 90%	General hospital, Spain May 2010 – September 2014	Stroke *Inclusion:* consecutive patients admitted to hospital confirmed stroke diagnosis *Exclusion:* previous diagnosis of OD; transient ischemic attack; transferred from another hospital *Time of assessment:* between 24 and 48 h after admission; before oral feeding	395 (*M* = 211; *F = *184) Mean age (SD): 73.2 (13.13)	*Oropharyngeal dysphagia (OD): any sign of impaired efficacy and/or safety of swallow; dysphagia*	*- Volume-Viscosity Swallow Test (V-VST)* (Clavé et al.) [74] Oropharyngeal dysphagia: signs and symptoms of impaired efficacy of swallow: -presence of oral residue (part of the bolus remaining in the oral cavity after swallow); -the efficiency of labial seal (ability to maintain the whole bolus in the oral cavity during the preparatory phase of swallow); -fractional swallow (multiple swallows per bolus) Signs of impaired safety of swallow: -changes in voice quality (including wet voice); cough; decrease in oxygen saturation ≥ 3% Tester: trained nursing staff	***Overall OD*** 45% (178/395)* *Oropharyngeal dysphagia per gender* *M* = 40.3% (85/211); *F = *50.5% (93/184)
Rösler et al. [46] J Am Med Dir Assoc	Prospective cohort AXIS 14/20; 70%	Teaching hospital, Germany Study period: NR	Dementia *Inclusion:* all consecutive patients admitted on acute geriatric ward with documented diagnosis of dementing illness with a minimum duration of 6 months *Exclusion:* patients with stroke within the previous 12 months; acute disease of the head or neck region; delirium according to Confusion Assessment Method (CAM) short form; intake of bensodiazepines on a regular basis; patients with dementia who were severely impaired in judgment according to neuropsychological evaluation without a legal caregiver; does not want to participate (4); transferred to another hospital (3), suffered stroke (1) *Time of assessment:* NR	161 (*M* = 44; *F = *117) Mean age (SD): 82.4 (NR)	*Dysphagia: difficulty of swallowing; signs of aspiration* *NB define dysphagia as aspiration*	*- Albertinen Dementia* *Dysphagia Screening (ADDS*): a self-composed screening for the risk of aspiration. Combined a water-swallowing test (DePippo et al.; Kidd et al.; Suiter and Leder) [94, 110, 111], pulse oximetry (Zaidi et al.) [112] and a test of different food consistencies (Trapl et al.) [98] Dysphagia: signs of aspiration -presence of at least 2 of the following symptoms: coughing or changes of voice (wet or hoarse vocal quality); a drop in oxygen saturation by ≥ 2% Tester: 2 experienced speech therapists trained with ADDS	***Overall Dysphagia*** ***WST*** 35.6% (57/160; n_missing_ = 1)* ***Overall dysphagia with apple slice*** 15.1% (22/144; n_missing_ = 17); ***Overall dysphagia with apple puree*** 6.3% (10/159; n_missing_2)
Sarabia-Cobo et al. [69] Appl Nurs Res	Retrospective cohort (III-2) AXIS 18/20; 90%	Nursing homes (12), Spain 2011 -2013	Mixed (dementia, cerebrovascular disease, pneumonia, bronco-aspiration) *Inclusion:* Dementia, cerebrovascular disease, pneumonia *Exclusion:* NR *Time of assessment:* NR	2384 (*M* = 635; *F = *1749) Mean age (SD); (95% CI; range): 88.7 (6.8); (82.1–89.60; 69–101)	*Oropharyngeal dysphagia: difficulty swallowing- subjective feeling of difficulty when passing food or liquid from the mouth and esophagus to the stomach; dysphagia*	*- Medical record review of:* - *Eating Assessment Tool-10* *(EAT-10)* (Belafsky et al.) [75] and - *3 oz Water Swallow Test* (DePippo et al.) [94] Oropharyngeal dysphagia: EAT-10 score ≥ 3; wet/hoarse voice quality after swallowing Tester: doctor from each center	***Overall OD*** 69.6% (1659/2384) *OD per gender* *M* = 73% (463/635); *F = *68.4% (1196/1749) *Subgroup OD with NGT or PEG (1659/2348)* *OD with NGT* 16.8% (278/1659); *OD with PEG dysphagia* 9.4% (156/1659) *NB Unclear prevalence calculation (i.e., fail one or both tests)*
Spronk et al. [47] Neurogastroenterol Motil	Prospective cross-sectional (IV) AXIS 15/20; 75%	General hospitals (2), Netherlands November 2017-February 2018	Mixed (cardiology, surgery, internal medicine, pulmonology, geriatrics, neurology, gastroenterology) *Inclusion:* All patients ≥ 18 years, admitted to surgical, internal medicine, gastroenterology, pulmonology, neurology, cardiology and geriatric wards *Exclusion:* no consent, admission duration ≥ 72 h or discharged before researcher could talk to them, cognitive dysfunction and /or delirium, language barriers, no oral intake permitted due to medical reasons or server impairment, severe illness with associated weakness to participate in study, admission to intensive care unit before general ward, unwillingness o shave off beard, as that prevented participation in sEMG signals, patient in contact isolation for infectious reasons *Time of assessment:* 24–72 h	205 (*M* = 108; *F = *97) Mean age median (Q1;Q2): 71 (NR) Age range: (60–78)	Dysphagia; swallowing problems, suspected dysphagia	*- Eating Assessment Tool (EAT-10)* (Belafsky et al.) [75] Score of ≥ 2 used as indication of swallowing problems *- Volume-Viscosity Swallow Test (V-VST)* (Clavé et al. 2008) [74] Tester: NR	***Overall dysphagia abnormal V-VST*** 7.3% (15/205)* ***Overall dysphagia EAT-10 score*** 23.4% 48/205 ***Overall suspected dysphagia determined by abnormal EAT-10 or V-VST*** 30.7% (63/205) *NB – inconsistencies in terminology usage for prevalence*
Stipancic, et al. [48] Am J Speech Lang Pathol	Prospective cross-sectional AXIS 13/20; 65%	University hospital, USA Study period: NR	Stroke *Inclusion*: first time ischemic stroke, ≥ 18 years, alert and responsive enough to partake in evaluations *Exclusion:* history of oropharyngeal dysphagia, have a previous disorder known to be associated with dysphagia, non-English speakers, patients recently intubated *Time of assessment*: up to 57.5 h after admission	100 (*M* = 63; *F = *27) Mean age (SD): 72.33 (14.4)	*Dysphagia; disordered swallowing*	*- Standardized clinical swallowing protocol* Patients with sign/symptoms of dysphagia during clinical swallowing evaluation completed videofluoroscopic swallow study (VFSS) or fiberoptic endoscopic evaluation of swallowing (FEES) Dysphagia: impairment in any of the phases of swallowing; oral, oropharyngeal, pharyngeal, or phayngoesophageal, identified by either clinical or instrumental evaluation Tester: speech-language pathologist	***Overall dysphagia*** 32% (32/100) (95% CI: 23, 41)* *NB No data reported from VFSS or FEES*
Sugiyama et al. [26] Geriatr Gerontol Int	Retrospective cross-sectional AXIS 13/20; 65%	Randomly selected: Nursing homes (1517), Long-term care facilities (941), Sanatorium medical facilities (1134), Rehabilitation hospitals (742), Japan 1.September-30.October 2009	NR *Inclusion:* Facility size and region *Exclusion:* facilities with fewer than 30 residents *Time of assessment:* NR	Nursing homes 440 (*M* = NR; *F = *NR) Mean age (SD): 85.9 (1.9) Long-term care facilities 275 (*M* = NR; *F = *NR) Mean age (SD): 84.8 (2.0) Sanatorium medical facility 204 (*M* = NR; *F = *NR) Mean age (SD): 80.8 (3.8) Rehabilitation hospitals 217 (*M* = NR; *F = *NR) Mean age (SD): 74.4 (4.8)	*Swallowing difficulty: oral feeding using thickened liquid diet, choking with meal intake, and current or past history of aspiration pneumonia and swallowing problems*	*- Standardized questionnaire* (number of residents): - use of feeding tubes - transitioning from tube feeding to oral intake - presence of swallowing problems Tester: institution dietician, nurse or other medical staff	***Overall swallowing difficulties among orally-fed residents per 100 beds*** Nursing homes n_missing_ = 34 23.7% (17.0?) (97/406); Long-term care facilities n_missing_ = 1: 15.6% (13.9?) (43/274); Sanatorium medical facilities n_missing_ = 1: 19.2% (24.7?) (39/203); Rehabilitation hospitals n_missing_ = 4: 15.4% (15.9?) (33/213) *Subgroup Tube fed patients per 100 beds* *Nursing homes* *11.6% (8.5) (51/440);* *Long-term care facilities 7.4% (7.0) (20/275);* *Sanatorium medical facilities* *36.3% (22.7) (74/204);* *Rehabilitation hospitals* *7.9% (7.4) (17/217)* *NB Standard deviations appear large*
Tanigör and Eyigör [33] European Geriatric Medicine	Prospective cross-sectional AXIS 10/20; 50%	Rehabilitation inpatient clinic, Turkey Study period: NR	Mixed (neurological, musculoskeletal and rheumatic diseases) *Inclusion:* adult inpatient, comply with instructions, given consent *Exclusion:* severe cognitive dysfunction, severe comorbidity or medical emergency impeding evaluation, delirium and end-stage cancer patient *Time of assessment:* first week of hospitalization	128 (*M* = 53; *F = *75) Mean age (SD): 56.5 (NR)	*Oropharyngeal dysphagia risk, swallowing difficulties*	*- History about eating habits, ev. difficulties with different consistencies/textures, severity rating (mild, moderate, severe) or associated signs (choking, wet voice, drooling xerostomia, mucositis, globus sensation, and dominant type of feeding (oral, nasogastric, gastro/jejunostomy)* * - Functional Oral Intake Scale (FOIS)* (Crary et al.) [113], - *Eating Assessment Tool - (EAT-10)* (Belafsky et al.) [75], - *MD Anderson Dysphagia Inventory (MDADI)* (Chen et al.) [89] Dysphagia: FOIS score ≤ 5; EAT-10 score ≥ 3, MDADI score (NR) Tester: NR *NB: Unclear criteria for determining dysphagia*	***Overall swallowing difficulties*** 22.6% (29/128) *NB: Unclear which combination of screening tests were used to determine prevalence; one, two or all*
van der Maarel-Wierink et al. [70] Int J Nurs Stud	Retrospective cross-sectional (IV) AXIS 16/20; 80%	Long-term care homes (119/360), Netherlands April 13 and November 2, 2010	Mixed (psychological disorder, dementia, cerebrovascular disease, other nervous system disorders, cardiovascular disease, respiratory disease/disorder, diabetes mellitus) *Inclusion:* data of care home residents ≥ 65 years *Exclusion:* tube fed patients *Time of assessment*: NR	8119 (*M* = 2116; *F = *6003) Mean age (SD): 84 (7.0) Age group: 65–75 (978/8119); 76–85 (3348/8119); > 85 (3793/8119)	*Subjective dysphagia; dysphagia; swallowing impairment: a symptom which refers to difficulty or discomfort during the progression of the bolus from the oral* cavity to the stomach	*- Data from the Dutch National Prevalence Survey of Care Problems*: -subjective dysphagia self-report (dichotomous scale: yes/no swallowing problems); -if self-report not possible: ward care provider report using similar scale or resident’s file check for swallowing complaints and/or dysphagia Tester: trained coordinator at each of care homes instruct care providers; two care providers	***Overall subjective dysphagia*** 9% (751/8119) *Subjective dysphagia per gender* *M* = 10% (214/2116); *F = *8.9% (537/6003) *Subjective dysphagia per age group* 65–75 16.8% (126/978); 76–85 9.9% (332/3348); > 85 7.7% (293/3793)
Vidal Casariego et al. [71] Nutrición Hospitalaria	Prospective cross-sectional (IV) AXIS 16/20; 80%	University hospital, neurology and internal medicine wards, Spain January-April 2019	Mixed acute illness *Inclusion:* > 18 years; urgent admission to neurology and internal medicine units *Exclusion:* hospitalized < 24 h, admitted to neurology or internal medicine unit but in charge of other hospital services; terminal stage of disease; expected death in following hours *NB no information on disease types* *Time of assessment:* NR	196 (*M* = 94; *F = *102) Mean age (SD): 74.4 (17.5)/76.0 (17.9)	*Dysphagia*	*- Eating Assessment Tool (EAT-10*) (Burgos et al.) [105] *NB No reference to original EAT-10* Tester: researchers	***Overall dysphagia*** 26.6% (42/158; n_unable to screen_ = 38)* *Dysphagia per gender* M = 33.3% (14/76); *F = *66.7% (28/82)
Wham et al. [49] Australas J Ageing	Prospective cross-sectional (IV) AXIS 14/20; 70%	Hospital and Residential care, New Zealand April – July 2014	NR *Inclusion:* ≥ 65 years (European ethnicity) or ≥ 55 years (Mãori and Pacific); able to understand and give consent, undertake a questionnaire and anthropometric measures *Exclusion:* participants with any known dysphagia risk *Time of assessment:* NR	Hospital: 57 (*M* = 23; *F = *34) Mean age (SD, range): 82.07 (6.92, 66.0–95.0) Residential care: 53 (*M* = 23; *F = *30) Mean age, (SD, range): 87 (6.65, 65.0–103.0)	*Swallowing difficulties; dysphagia; deglutitive disorders, swallowing problems; dysphagia risk*	*- Eating Assessment Tool (EAT-10)* (Schindler et al.) [114] Dysphagia risk: EAT-10 scores ≥ 3 Tester: NR	***Overall dysphagia risk per clinical setting:*** ***Overall in hospital*** 15.8% (9/57); ***Overall in residential care*** 32.1% (17/53)
Young and Durant-Jones [34] Dysphagia	Retrospective cohort (III-2) AXIS 10/20; 50%	Hospital, USA 3 years	Stroke *Inclusion:* randomly selected patients with cerebral vascular accident (CVA); non-comatose: at least 18 years of age; hospital stay at least 7 days; no previous history of CVA; no previously reported neurological disease, including dysphagia *Exclusion:* comatose patients (9) *Time of assessment:* NR	225 (*M* = NR; *F = *NR) Mean age (SD): NR	*Dysphagia: any oral or pharyngeal stage*, neuromuscular dysfunction that affected patients' ability to eat orally	*- Patients identified by ICD-9 codes and chart review performed:* -identified symptoms of dysphagia via a standardized form -physician bedside assessment: presence or absence of the gag reflex Tester: chart review by 2 speech-language pathologists	***Overall dysphagia*** 28% (65/216; n_comatosed_ = 9) *Suspected aspiration:* 25.5% (55/216; n_comatosed_ = 9); *Aspiration pneumonia:* 11.1% (24/216; n_comatosed_ = 9); *Choking:* 4.1% (9/216; n_comatosed_ = 9); *Coughing:* 13.4% (29/216; n_comatosed_ = 9); *Reduced gag reflex:* 21.8% (47/216; n_comatosed_ = 9) *NPO status*: 24.5% (53/216; n_comatosed_ = 9); *NG tube*: 17.1% (37/216; n_comatosed_ = 9); *G-tube:* 3.7 (8/216; n_comatosed_ = 9)

^a^ NHMRC hierarchy: Level 1 Systematic reviews; Level II Prospective cohort study; Level III–1 All or none; Level III–2 Retrospective cohort; Level III–3 Case–control study; Level IV Cross-sectional study or case series;

NR: not reported; NB: please note

^*^Data included in the meta-analysis for hospital setting; **Data included in the meta-analysis for rehabilitation setting; ***Data included in the meta-analysis for nursing home setting

### Study Characteristics

All extracted data are summarized in Table 2. Data were recorded under eight subheadings: author, journal, study design, AXIS score, study setting, country and time period, underlying medical diagnosis, inclusion/exclusion criteria and time of assessment, sample characteristics (sample size, gender, age in years), description of OD terminology used in the study, screening/assessment tools used for prevalence calculation and what professions completed the testing, and OD prevalence data.

The included studies were published from 1986 to 2020, the majority (36/45) after 2010. The studies originated from 23 countries; 27 from Europe [25, 27, 28, 39, 41–47, 51, 52, 55, 57, 58, 61–66, 68–72], six from North America [34, 40, 48, 56, 59, 60], three from Oceania [49, 53, 54], four from Asia [26, 33, 37, 67], two from South America [36, 50], and two from Africa [35, 38]. Twenty-nine studies were prospective study designs: ten cohort and 19 cross-sectional designs. Fourteen studies were retrospective: eight cohort and six cross-sectional. One study included both retrospective and prospective cross-sectional data.

There were 32 articles providing estimates for OD prevalence from hospitals [26, 28, 34–36, 38–40, 42, 43, 45–57, 60–66, 68, 71], four from rehabilitation [26, 33, 58, 59], and 12 from nursing home settings [25–28, 37, 41, 44, 49, 67, 69, 70, 72]. Two articles provided OD prevalence estimates from both hospital and nursing home settings [28, 49] and one article reported OD prevalence estimates from all three settings: hospital, rehabilitation, and nursing home [26]. There were no studies that met the inclusion criteria from palliative care.

### Healthcare Setting Description

The description of the hospital settings in this systematic review included general, tertiary, teaching, and regional hospitals. Hospital wards such as acute care, medical, neurological, and geriatric were used for participant recruitment. The rehabilitation settings included an inpatient rehabilitation clinic, rehabilitation facilities, and hospital (neuro)rehabilitation services/units. Settings that were classified as nursing homes included short-term/intermediate care, residential care, long-term care, and assisted living [73].

### Participants

An estimated total of 49,436 participants were included in the 44 studies; 24,309 from hospitals, 541 from rehabilitation, and 24,586 from nursing homes. The number of participants per study ranged from 49 to 14,913, with a median participant number of 228 (25th percentile 143; 75th percentile 438). Forty-two studies, consisting of 48 datasets, included participants with a mean age of 75 years (SD 10; range 54–106 years). Two studies did not report ages, but specified the population as adult or geriatrics. The majority of studies included participants with stroke (*n* = 19) [34–36, 38, 39, 42, 48, 51–54, 56–60, 62, 66, 68]. Fifteen studies included patients with diverse diagnoses: e.g., post-surgery, internal medicine, geriatrics, pneumonia, trauma, gastrointestinal tract disease, cardiovascular disease, respiratory tract disorders, fractures, musculoskeletal, neurologic and neurodegenerative diseases, and head and neck cancer [25, 27, 28, 33, 40, 43, 45, 47, 50, 55, 61, 63, 69–71]. One study included only participants with dementia [46] and nine studies did not specify the participants’ diagnosis [26, 37, 41, 44, 49, 64, 65, 67, 72].

### Type of Screening or Assessment Method

The type and combination of screening and assessment methods used to determine the prevalence for OD varied. Nearly one-third (15/44) of the studies [25, 35, 38, 39, 46, 51, 55, 57, 61–65, 67, 68] used a screening tool alone to identify risk of OD and three studies [36, 56, 66] used clinical non-instrumental assessments to diagnose OD. Four studies used FEES and/or VFSS either in combination with a screening [42], a clinical non-instrumental assessment [48, 58, 59]. Five studies used a patient-reported outcome measure (PROM) alone [37, 43, 49, 50, 71], three studies used a PROM together with a screen [44, 47, 69], and one used two PROMs and a clinical swallowing assessment [33]. Eight studies determined OD through a chart review [34, 40, 45, 52–54, 60, 72] five studies used self-formulated questions to staff and/or patient [26–28, 41, 70].

Over half (26/44) of studies reported OD prevalence data using screening and clinical non-instrumental assessments methods or tools that were either designed by the authors for the purpose of the study or modified versions of published tools, thus lacking information on diagnostic performance and psychometric properties [26–28, 34, 36, 38–42, 44–46, 48, 52–54, 58–60, 62, 64–66, 70, 72]. Of the screening and clinical non-instrumental assessment tools used to determine OD with known diagnostic performance and psychometric properties, the Volume-Viscosity Swallow Test (V-VST) [74] was the most commonly used screen [47, 55, 61, 63, 64, 68] and the Eating Assessment Tool-10 (EAT-10) [75] was the most frequently used PROM [33, 37, 43, 47, 49, 50, 69, 71]. The Mann Assessment of Swallowing Ability (MASA) [76] was the only clinical swallowing assessment used with known psychometric characteristics (one study) [56]. Table 2 provides an overview of the screens and assessments used in all of the included studies.

### Time of Screening or Assessment

The time of screening or assessment for OD prevalence was recorded in 24/44 of the included studies. OD prevalence estimates from the hospital setting (21/32) were either reported as time post-stroke (7/21) or time from admission (14/21). Time post-stroke ranged from hyperacute phase [77]; ≤ 24 h post-stroke (1/7) [66], to acute phase; 1–7 days (4/7) [35, 42, 53, 60], to early subacute phase; 7 days–3 months (2/7) [36, 39]. Moment of screening or assessment in the rehabilitation setting was reported as hours or days from admission in three studies [33, 58, 59] and one study [26] did not specify when the participants were screened or assessed in relation to onset of disease or illness. None of the studies from the nursing home setting [25–28, 37, 41, 44, 49, 67, 69, 70, 72] specified the moment of screening or assessment for OD prevalence (Table 2).

### Meta-analyses

In accordance with the pre-defined criteria, twelve studies that included data collected from medical records, national databases, surveys, or registries [26, 34, 40, 43, 45, 50–54, 60, 72] were excluded from meta-analyses. Further, four studies that used nurses,’ patients,’ or caregivers’ responses to a single dichotomous question about the presence of swallowing difficulties as screen for OD prevalence [27, 28, 41, 70] were also not included in the meta-analysis. In addition, six studies were excluded due to the inability to compute proportional OD prevalence data results from the datasets [33, 44, 49, 64, 65, 69]. The remaining 22 studies, 17 from the hospital, 2 from rehabilitation and 3 from nursing home settings, were included in the meta-analysis. Studies used screening [25, 35, 38, 39, 46, 55, 57, 61–63, 67, 68], PROM [37, 71], clinical non-instrumental assessments [36, 56, 66], or a combination of methods (screen, clinical swallowing assessment, PROM, instrumental) [42, 47, 48, 58, 59]. One study provided OD prevalence data for both screening and patient self-report for the entire study population [47]. As screening was preferred over self-report data, only prevalence estimates based on screening were included in the meta-analysis. Table 2 provides an overview of prevalence estimates as retrieved from individual studies; data used for meta-analyses have been marked.

### Hospital

**Fig. 2:**
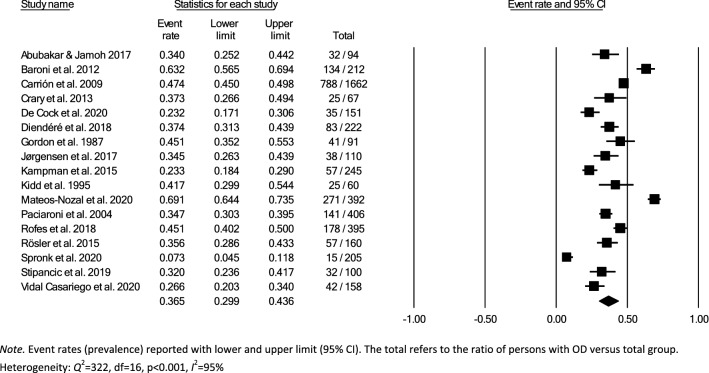
Random-effects forest plot for overall pooled OD prevalence estimate in the hospital setting

**Fig. 3:**
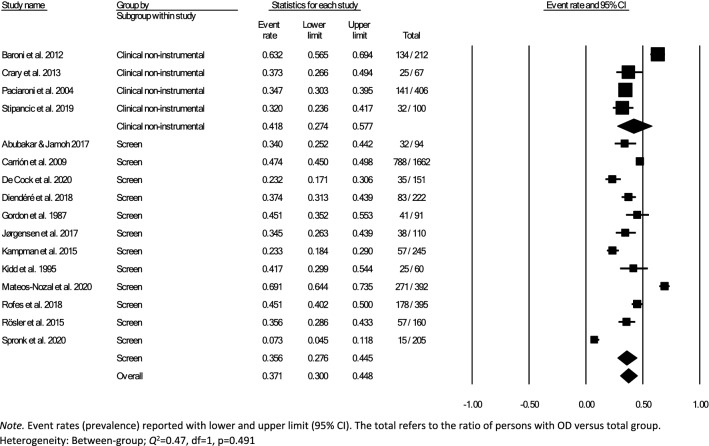
Random-effects forest plot for OD prevalence in hospital setting; between-group screen and clinical non- instrumental assessment

**Fig. 4:**
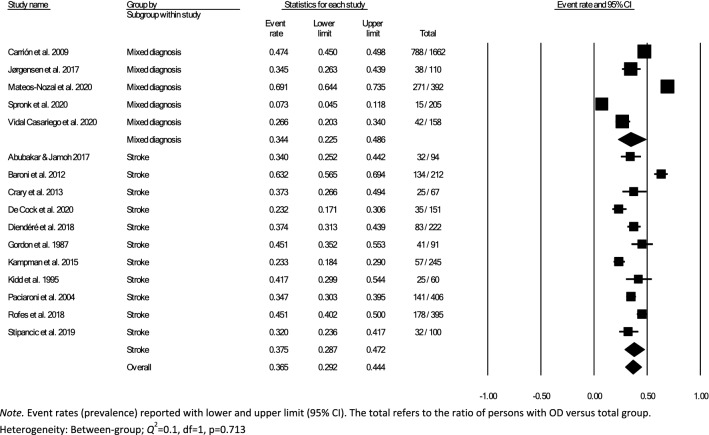
Random-effects forest plot for OD prevalence in hospital setting; between-group stroke and mixed diagnoses

**Fig. 5:**
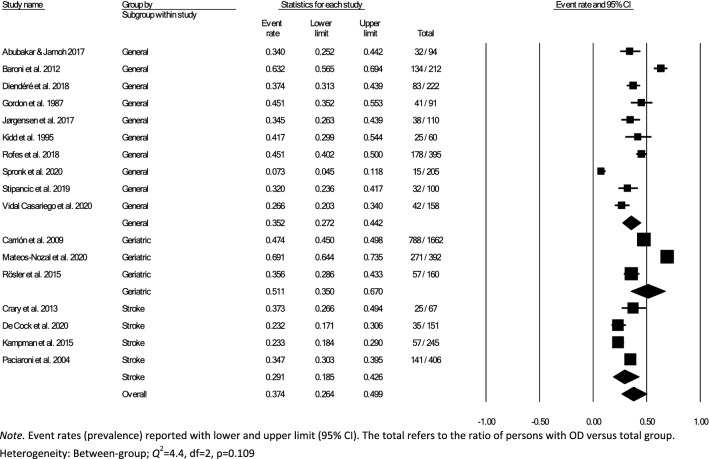
Random-effects forest plot for OD prevalence in hospital setting; between-group type of ward

Meta-analysis using OD prevalence data from 17 hospital studies [35, 36, 38, 39, 42, 46–48, 55–57, 61–63, 66, 68, 71] resulted in an overall pooled OD prevalence estimate of 36.5% (95% confidence interval [CI] 29.9 − 43.6) (Fig. 2). Between-group analysis was computed for type of assessment (screen versus clinical assessment), diagnosis and type of ward. Twelve studies used screening [35, 38, 39, 42, 46, 47, 55, 57, 61–63, 68] and four studies used clinical non-instrumental assessment [36, 48, 56, 66] resulting in pooled OD prevalence estimates of 35.6% (95% CI 27.6 − 44.5) and 41.8% (95% CI 27.4 − 57.7), respectively (Fig. 3). Eleven studies included stroke diagnosis [35, 36, 38, 39, 42, 48, 56, 57, 62, 66, 68] and five studies included mixed diagnosis [47, 55, 61, 63, 71] resulting in pooled OD prevalence estimates of 37.5% (95% CI 28.7–47.2) and 34.4% (95% CI 22.5–48.6), respectively (Fig. 4). A meta-analysis for type of ward in the hospital setting revealed estimated OD prevalence of 35.3% (95% CI 27.2–44.2) for general or non-specified wards (10/17) [35, 36, 38, 39, 42, 47, 48, 61, 68, 71], 29.1% (95% CI 18.5–42.6) for stroke wards (4/17) [56, 57, 62, 66], and 51.1% (95% CI 35.0–67.0) for geriatric wards 3/17) [46, 55, 63] (Fig. 5). None of the between-group differences were significant. This meta-analysis incorporates data from 17 studies, which yield a *z*-value of − 12.00171 and corresponding 2-tailed *p*-value < 0.001. The fail-safe N is 621. This means that we would need to locate and include 621 “null” studies in order for the combined 2-tailed *p*-value to exceed 0.050.

#### Rehabilitation

**Fig. 6:**
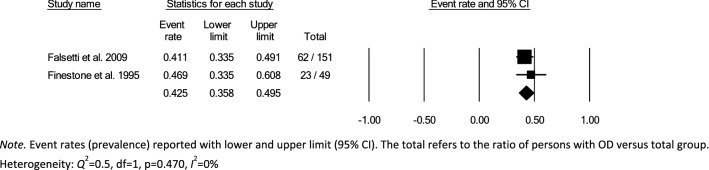
Random-effects forest plot overall OD prevalence in rehabilitation setting

The two included rehabilitation studies [58, 59] used clinical non-instrumental assessments revealing an estimated overall pooled prevalence for OD of 42.5% (95% CI 35.8–49.5) (Fig. 6). This meta-analysis of the prevalence of OD in the rehabilitation setting used data from two studies only, thus a fail-safe N analysis for publication bias was not available.

#### Nursing Home

**Fig. 7:**
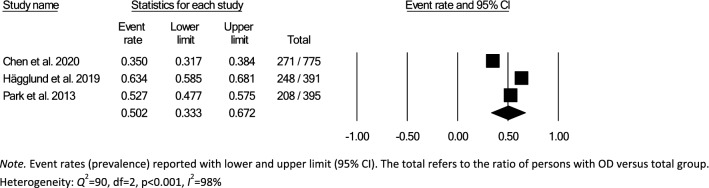
Random-effects forest plot overall OD prevalence nursing home setting

**Fig. 8:**
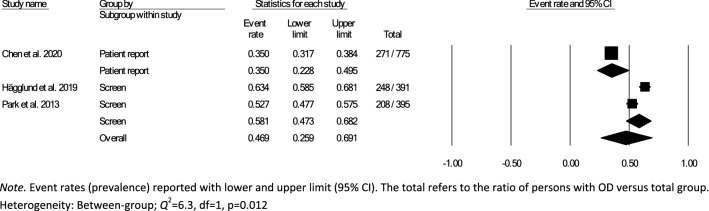
Random-effects forest plot for OD prevalence in nursing home; between-group screening and patient-report outcome measure

Three studies from nursing homes [25, 37, 67] revealed estimated overall pooled OD prevalence of 50.2% (95% CI 33.3–67.2) (Fig. 7). Two of the three studies [25, 67] used screenings and one used a PROM, resulting in an estimated pooled OD prevalence of 58.1% (95% CI 47.3–68.2) and 35.0% (95% CI 22.8–49.5), respectively (Fig. 8). Total between-group OD prevalence estimates were significant (*p* = 0.012). This meta-analysis incorporates data from 3 studies, which yield a *z*-value of − 1.11840 and corresponding 2-tailed *p*-value of 0.263. Since the combined result is not statistically significant, the fail-safe N (which addresses the concern that the observed significance may be spurious) is not relevant.

## Discussion

### Systematic Review Findings

This systematic review and meta-analysis were conducted to establish the estimated pooled prevalence for OD in adults in different healthcare settings. The majority of the 44 included studies represented hospital (32/44) and nursing home (12/44) settings. There were few studies identified from the rehabilitation setting (4/44) and none from palliative care, revealing a knowledge gap regarding OD prevalence in these settings. Studies in the hospital and rehabilitation settings dated from 1986 to 2020, whereas the nursing home studies were from 2013 to 2020, possibly reflecting an increased awareness and exploration of OD in the elderly and nursing home population in the past decade.

Estimations of OD prevalence are dependent on (a) the definition of OD used in studies; (b) the choice of measure (screen, clinical non-instrumental assessment, instrumental assessment, or patient-reported outcome measurement); (c) the diagnostic performance and psychometric properties (including validity, reliability, responsiveness) and feasibility of the chosen measure; and (d) time of assessment (e.g., during the acute or chronic phase of the underlying disease) [13, 20, 78]. In this systematic review, terminology and definitions of oropharyngeal dysphagia varied and were conflicting. The majority of studies used the general term “dysphagia” in reporting prevalence of swallowing disorders, some studies referred to OD but lacked defining the concept of OD, while many studies provided a broad, generic definition of what constituted OD (e.g., lower capacity to swallow, generally unsafe swallow). Other studies included a definition that comprised both oropharyngeal and esophageal phases of swallowing (e.g., difficulty moving a bolus from the mouth to the stomach) or included aspects outside of OD (e.g., sitting position, difficulty transferring of food to the mouth, appetite) in addition to OD-related aspects. A consensus on a universal definition of dysphagia in the reporting of OD prevalence would support a more accurate estimation of OD prevalence [13].

In addition to lacking a universal definition for dysphagia, this systematic review revealed inconsistencies in the literature regarding what constitutes a screening and clinical non-instrumental assessment tool when estimating the prevalence of OD [14, 79, 80]. Several studies in this systematic review reported OD prevalence data from “screening”, but used measures that included administering a variation of food and liquid volumes and consistencies. This differs from common definitions of screenings: screenings for OD are designed to ensure identification of persons at risk of dysphagia and determine the need for further assessment, whereas clinical non-instrumental assessments are designed to ascertain the presence, location, severity, and possible treatment of OD [13]. The level of diagnostic performance of screening tools and psychometric robustness of clinical non-instrumental assessment methods chosen to estimate OD prevalence in the included studies is of high importance. Several systematic reviews have scrutinized the diagnostic performance and psychometric properties of available screening and clinical non-instrumental assessments [14, 79–84] indicating frequent poor methodological quality and lack of sufficient details. Instrumental assessments (VFSS or FEES) would be preferable when determining prevalence, as they have been shown to identify dysphagia in 20–30% more patients than screening and clinical non-instrumental assessments [20]. However, instrumental assessments require specialized training and equipment, thus, due to feasibility (e.g., availability, ease of administration), screenings and clinical non-instrumental assessments are the natural first choice for estimating the prevalence of OD [13, 85]. The most frequently used PROM in this systematic review used for estimating prevalence of OD was the EAT-10 (8/44) and five of these studies used the EAT-10 in isolation to screen for OD. This PROM was developed to assess symptom severity, quality of life, and treatment efficacy to be used for patients with both oropharyngeal and esophageal dysphagia [75]. However, in 2017, Cordier and colleagues evaluated the EAT-10 using Rasch analysis, challenging its diagnostic performance and psychometric properties, and recommended that it was re-developed using the Rasch model [86]. These findings were supported by other authors [80, 87, 88]. Furthermore, some studies used measurements in populations other than for which they were developed, which may affect the reliability and validity of the instrument [13]. For example, in a study by Tanigör and colleagues [33], the MDADI, developed to assess quality of life for patients with head and neck cancer [89], was used to determine the prevalence of OD in populations with neurological, musculoskeletal, and rheumatic diseases. This systematic review highlights the need for clinicians and researchers to use screening and assessment tools with optimal diagnostic performance and psychometric properties that are tailored for the population of interest when screening or assessing for OD.

### Meta-analysis Findings

The meta-analysis included half (22/44) of the studies included in this systematic review: 17 from hospitals, two from rehabilitation, and three from nursing home settings. Results revealed an overall estimated pooled OD prevalence of 36.5% (95% confidence interval [CI] 29.9 − 43.6) in the hospital setting. Three studies in the meta-analysis in the hospital setting showed relatively high [36, 63] and low [47] OD estimates as compared to the other studies. Baroni et al. studied OD prevalence in stroke patients, including those with previous stroke. This inclusion criterion was an exclusion criterion in several of the included studies reporting on OD prevalence in stroke. In addition, OD was determined through a clinical evaluation using a broad definition of OD, if one or more *“swallowing changes”* were observed [36]. Mateos-Nozal et al. studied OD prevalence in the acute geriatric population with inclusion criteria ≥ 80 years. OD was determined if the V-VST revealed “any sign of OD.” In contrast, Spronk and colleagues studied all general hospital admissions on several wards and used both the EAT-10 and V-VST to determine OD prevalence. Participants were judged positive for OD with an EAT-10 score of ≥ 2 and *“if a in any category of viscosity or multiple categories of viscosity, the maximum bolus volume was not reached”* on the V-VST [47]. This study chose to apply different values to define OD than described by the original validation studies for the EAT-10 [75] and V-VST [74]. In this study, the low OD prevalence results for the V-VST were used in the meta-analysis as it is considered as having better evidence base for determining the presence of OD than the EAT-10. Although each of these individual studies did not have an impact on the estimate of overall pooled OD prevalence in the hospital setting, variations in study design, sample population, and definitions of OD provide insight into the heterogeneity of the included studies.

An overall estimated pooled OD prevalence of 42.5% (95% CI 35.8–49.5) was established from two studies in the rehabilitation setting. It might be expected that the estimated pooled OD prevalence in the hospital setting would be higher than the rehabilitation setting due to the acuteness of the underlying disease. The hospital setting included twice as many studies with stroke patients (11/17) than those with mixed diagnosis (5/17) and swallowing function tends to resolve in many patients within the first few days following stroke [90, 91]). Thus, patients with persisting OD often present with a more severe sequelae [92, 93] and require rehabilitation. In addition, the majority of studies from the hospital setting used screening tools to determine OD prevalence whereas the two studies from (neuro)rehabilitation unit/service, located within a hospital, utilized clinical non-instrumental methods and/or instrumental assessments to identify OD prevalence. Previous research has shown that use of screening methods results in lower prevalence than using clinical assessments [1, 20]. It is concerning that so few prevalence studies were identified from the rehabilitation setting. There is a need for future prevalence studies from the rehabilitation population.

As expected, nursing home settings revealed the highest OD prevalence; three studies showed an overall estimated pooled prevalence of 50.2% (95% CI 33.3–67.2) and an even higher estimated pooled OD prevalence of 58.1% for between-group difference for two of the three studies that used screening tools. Populations in the nursing home setting were older compared to other settings and suffered many medical conditions associated with OD (e.g., diseases of the circulatory and nervous systems, and cognitive disorders). Also, presbyphagia and sarcopenia may exacerbate OD resulting from comorbidities common to the aging population [6]. Results from the between-group analysis for type of ward also revealed a higher OD prevalence for the geriatric ward (51.1%), which was very similar to the nursing home setting. This systematic review reveals that overall pooled OD prevalence estimates are high for all healthcare settings, but highest in nursing homes.

The absence of studies in this systematic review included from the palliative setting raises concerns for this population. Patients receiving end of life care were excluded from six studies [33, 41, 62, 63, 65, 71] in this systematic review. This gives cause for concern regarding whether or not these patients are being screened or assessed for OD and how their OD is being managed. This systematic review has identified a need of further research in palliative healthcare setting.

### Limitations

This systematic review is not without its limitations. The literature search only included two of the most relevant databases and English publications, thus, giving rise to potential publication bias. Furthermore, meta-analysis is subject to heterogeneity in study design, study population, and choice of outcome measures. Included studies differed in the definition of OD, definition of screening compared to clinical assessment, methodological study quality, and diagnostic performance and psychometric properties of outcome measures were used to determine OD prevalence. The larger number of studies included mainly stroke populations and may, therefore, limit the generalizability of calculated prevalence estimates. Consequently, although measures were taken to reduce heterogeneity for studies included in the meta-analysis, caution should be used when interpreting the results.

## Conclusion

This systematic study reviewed 44 articles reporting on the prevalence of OD in different healthcare settings (hospital, rehabilitation, and nursing home). Most studies were conducted in hospital and nursing home settings, few studies in rehabilitation, and no studies were identified that reported on palliative care facilities. Future prevalence studies should provide data especially for patients in rehabilitation and palliative care. Overall, pooled prevalence estimate for OD determined by meta-analysis was high for all healthcare settings. Results revealed an overall estimated pooled OD prevalence of 36.5 (95% CI 28.8 − 44.9) for the hospital setting, 42.5% (95% CI 39.9–53.4) for the rehabilitation setting, and 50.2% (95% CI 33.3–67.2) for the nursing home setting. These high OD prevalence estimates across healthcare settings indicate that there is a large number of people at risk for malnutrition, dehydration, aspiration pneumonia, and ultimately a reduced quality of life. These findings indicate that treatment pathways including early assessment and diagnosis of OD should be a priority for healthcare professionals working in different healthcare settings with populations at risk for OD. In addition, this systematic review emphasizes a need for consensus in OD-related terminology and use of a clear operational definition when reporting OD prevalence. Further, when choosing screening and assessment tools to identify and assess OD, clinicians and researchers should take the target population into account for which a measure has been developed and validated, as well as only select screening and assessment tools with optimal diagnostic performance and psychometric properties.

## Supplementary Information

Below is the link to the electronic supplementary material.

Supplementary file1 (PDF 682 kb)
