# Supplementary material for: Prevalence of Oropharyngeal Dysphagia in Adults in Different Healthcare Settings: A Systematic Review and Meta-analyses
Source: Dysphagia. 2022 May 31;38(1):76–121. doi: 10.1007/s00455-022-10465-x (PMC9873728; doi:10.1007/s00455-022-10465-x)
Supplement: Supplementary file 1 — Supplementary file1 (PDF 682 kb) [file 455_2022_10465_MOESM1_ESM.pdf]

**Article title:** Prevalence of oropharyngeal dysphagia in adults in different healthcare settings: A systematic review and meta-analyses

**Journal name:** Dysphagia

**Author names:** Maribeth Caya Rivelsrud, Lena Hartelius, Liza Bergström, Marianne Løvstad, Renée Speyer

**Affiliation and name of corresponding author:** Sunnaas Rehabilitation Hospital, maribeth.rivelsrud@sunnaas.no

**Online Resource 1.** Methodological quality assessment of studies using AXIS [1]

| Author (alphabetical order) | Q1 | Q2 | Q3 | Q4 | Q5 | Q6 | Q7 | Q8 | Q9 | Q10 | Q11 | Q12 | Q13 | Q14 | Q15 | Q16 | Q17 | Q18 | Q19 | Q20 | Total |
|---|---|---|---|---|---|---|---|---|---|---|---|---|---|---|---|---|---|---|---|---|---|
| Abubakar et al., 2017 [2] | 1 | 1 | 0 | 1 | 1 | 0 | 0 | 1 | 1 | 1 | 1 | 1 | 0 | 0 | 1 | 1 | 1 | 1 | 1 | 1 | 15 |
| Andrade et al., 2018 [3] | 1 | 1 | 0 | 1 | 1 | 1 | 0 | 1 | 1 | 1 | 1 | 1 | 1 | 0 | 1 | 1 | 0 | 1 | 1 | 1 | 16 |
| Arnold et al., 2016 [4] | 1 | 1 | 0 | 1 | 1 | 1 | 1 | 1 | 1 | 1 | 1 | 1 | 1 | 0 | 1 | 1 | 1 | 1 | 1 | 1 | 18 |
| Baroni et al., 2012 [5] | 1 | 1 | 0 | 1 | 1 | 1 | 0 | 1 | 1 | 1 | 1 | 1 | 0 | 1 | 1 | 1 | 1 | 0 | 0 | 1 | 15 |
| Beharry et al., 2019 [6] | 1 | 1 | 0 | 1 | 1 | 1 | 0 | 1 | 1 | 1 | 1 | 1 | 0 | 0 | 1 | 1 | 1 | 1 | 1 | 1 | 16 |
| Blanař et al., 2019 [7] | 1 | 1 | 0 | 1 | 1 | 1 | 1 | 1 | 0 | 1 | 1 | 1 | 1 | 1 | 1 | 1 | 1 | 1 | 1 | 1 | 18 |
| Brogan et al., 2014 [8] | 1 | 1 | 0 | 1 | 1 | 1 | 0 | 1 | 1 | 1 | 1 | 1 | 0 | 1 | 1 | 1 | 1 | 1 | 1 | 1 | 17 |
| Brogan et al., 2014 [9] | 1 | 1 | 0 | 1 | 1 | 1 | 0 | 1 | 1 | 1 | 1 | 1 | 0 | 1 | 1 | 1 | 1 | 1 | 1 | 1 | 17 |
| Carrión et al., 2015 [10] | 1 | 1 | 0 | 1 | 1 | 1 | 1 | 1 | 1 | 1 | 1 | 1 | 1 | 0 | 1 | 1 | 1 | 1 | 1 | 1 | 18 |
| Chen et al., 2020 [11] | 1 | 1 | 0 | 1 | 1 | 1 | 0 | 0 | 0 | 0 | 1 | 1 | 0 | 0 | 1 | 1 | 1 | 1 | 1 | 1 | 13 |
| Crary et al., 2013 [12] | 1 | 1 | 0 | 1 | 1 | 1 | 0 | 1 | 1 | 1 | 1 | 1 | 1 | 0 | 0 | 1 | 1 | 1 | 1 | 1 | 16 |
| De Cock et al., 2020 [13] | 1 | 1 | 0 | 1 | 1 | 1 | 1 | 1 | 1 | 1 | 1 | 1 | 1 | 1 | 1 | 1 | 1 | 1 | 1 | 1 | 19 |
| Diendéré et al., 2018 [14] | 1 | 1 | 0 | 1 | 1 | 1 | 0 | 1 | 1 | 1 | 1 | 1 | 0 | 0 | 1 | 1 | 1 | 0 | 0 | 1 | 14 |
| Falsetti et al., 2009 [15] | 1 | 1 | 0 | 1 | 1 | 1 | 0 | 1 | 1 | 1 | 1 | 1 | 1 | 0 | 1 | 1 | 1 | 1 | 0 | 1 | 16 |
| Finestone et al., 1995 [16] | 1 | 1 | 0 | 1 | 1 | 1 | 1 | 1 | 1 | 1 | 1 | 1 | 0 | 1 | 1 | 1 | 1 | 0 | 1 | 1 | 17 |
| Flowers et al., 2013 [17] | 1 | 1 | 0 | 1 | 1 | 1 | 1 | 1 | 0 | 1 | 1 | 1 | 0 | 1 | 1 | 1 | 1 | 1 | 0 | 1 | 16 |
| Gordon et al., 1987 [18] | 1 | 1 | 0 | 1 | 1 | 1 | 1 | 1 | 0 | 1 | 1 | 1 | 1 | 1 | 1 | 1 | 1 | 0 | 0 | 0 | 15 |
| Groher and Bukatman, 1986 [19] | 1 | 1 | 0 | 1 | 1 | 1 | 0 | 1 | 0 | 0 | 1 | 1 | 1 | 0 | 1 | 1 | 1 | 0 | 0 | 0 | 12 |
| Hollaar et al., 2017 [20] | 1 | 1 | 0 | 1 | 1 | 1 | 1 | 1 | 0 | 1 | 1 | 1 | 1 | 0 | 1 | 1 | 1 | 1 | 1 | 1 | 17 |
| Huppertz et al., 2018 [21] | 1 | 1 | 0 | 1 | 1 | 1 | 0 | 1 | 0 | 1 | 1 | 1 | 0 | 0 | 1 | 1 | 1 | 1 | 1 | 1 | 15 |

| | | | | | | | | | | | | | | | | | | | | | |
|---|---|---|---|---|---|---|---|---|---|---|---|---|---|---|---|---|---|---|---|---|---|
| Hägglund et al., 2018 [22] | 1 | 1 | 0 | 1 | 1 | 0 | 0 | 1 | 1 | 1 | 1 | 1 | 0 | 0 | 1 | 1 | 1 | 1 | 1 | 1 | 15 |
| Jørgensen et al., 2017 [23] | 1 | 1 | 0 | 1 | 1 | 1 | 0 | 1 | 0 | 1 | 1 | 1 | 1 | 0 | 1 | 1 | 1 | 1 | 1 | 1 | 16 |
| Kampman et al., 2015 [24] | 1 | 1 | 0 | 1 | 1 | 1 | 0 | 1 | 1 | 0 | 1 | 1 | 0 | 0 | 1 | 1 | 1 | 1 | 1 | 0 | 14 |
| Kidd et al., 1995 [25] | 1 | 1 | 0 | 1 | 1 | 1 | 0 | 1 | 1 | 1 | 1 | 1 | 1 | 0 | 1 | 1 | 1 | 0 | 0 | 0 | 14 |
| Lindroos et al., 2014 [26] | 1 | 1 | 0 | 1 | 1 | 1 | 0 | 1 | 0 | 1 | 1 | 1 | 0 | 0 | 1 | 1 | 1 | 1 | 1 | 1 | 15 |
| Mañas-Martinez et al., 2018 [27] | 1 | 1 | 0 | 1 | 1 | 0 | 0 | 0 | 1 | 1 | 1 | 1 | 0 | 0 | 1 | 1 | 0 | 1 | 1 | 0 | 12 |
| Mateos-Nozal et al., 2020 [28] | 1 | 1 | 0 | 1 | 1 | 1 | 1 | 1 | 1 | 0 | 1 | 1 | 1 | 1 | 1 | 1 | 1 | 1 | 1 | 1 | 18 |
| Melgaard, Rodrigo-Domingo and Mørch, 2018 [29] | 1 | 1 | 0 | 1 | 1 | 1 | 1 | 1 | 1 | 1 | 1 | 1 | 0 | 1 | 1 | 1 | 1 | 1 | 1 | 1 | 18 |
| Nielsen et al., 2018 [30] | 1 | 1 | 0 | 1 | 1 | 0 | 1 | 1 | 1 | 1 | 1 | 1 | 0 | 1 | 1 | 1 | 1 | 1 | 1 | 1 | 17 |
| Nogueira and Reis, 2013 [31] | 1 | 1 | 0 | 1 | 1 | 0 | 0 | 0 | 0 | 1 | 1 | 1 | 1 | 0 | 1 | 1 | 0 | 0 | 1 | 0 | 11 |
| Paciaroni et al., 2004 [32] | 1 | 1 | 0 | 1 | 1 | 1 | 0 | 1 | 1 | 1 | 1 | 1 | 1 | 0 | 1 | 1 | 1 | 0 | 0 | 0 | 14 |
| Park et al., 2013 [33] | 1 | 1 | 0 | 1 | 1 | 0 | 0 | 1 | 1 | 1 | 1 | 1 | 0 | 1 | 1 | 1 | 1 | 1 | 1 | 1 | 16 |
| Patel and Martin, 2008 [34] | 1 | 1 | 0 | 1 | 1 | 0 | 1 | 0 | 0 | 1 | 1 | 1 | 0 | 1 | 1 | 1 | 1 | 1 | 0 | 1 | 14 |
| Rofes et al., 2018 [35] | 1 | 1 | 1 | 1 | 1 | 1 | 0 | 1 | 1 | 1 | 1 | 1 | 0 | 1 | 1 | 1 | 1 | 1 | 1 | 1 | 18 |
| Rösler et al., 2015 [36] | 1 | 1 | 0 | 1 | 1 | 0 | 1 | 0 | 0 | 1 | 1 | 1 | 1 | 1 | 1 | 1 | 0 | 0 | 1 | 1 | 14 |
| Sarabia-Cobo et al., 2016 [37] | 1 | 1 | 0 | 1 | 1 | 1 | 1 | 1 | 1 | 1 | 1 | 1 | 1 | 1 | 1 | 1 | 1 | 0 | 1 | 1 | 18 |
| Spronk et al., 2020 [38] | 1 | 1 | 0 | 1 | 1 | 1 | 0 | 1 | 1 | 1 | 1 | 1 | 0 | 0 | 0 | 1 | 1 | 1 | 1 | 1 | 15 |
| Stipancic et al., 2019 [39] | 1 | 1 | 0 | 1 | 1 | 0 | 0 | 1 | 1 | 0 | 1 | 1 | 0 | 0 | 0 | 1 | 1 | 1 | 1 | 1 | 13 |
| Sugiyama et al., 2014 [40] | 1 | 1 | 0 | 1 | 1 | 0 | 0 | 1 | 0 | 1 | 0 | 1 | 0 | 0 | 1 | 1 | 1 | 1 | 1 | 1 | 13 |
| Tanigör and Eyigör, 2020 [41] | 1 | 1 | 0 | 1 | 1 | 1 | 0 | 0 | 1 | 0 | 0 | 0 | 0 | 0 | 1 | 0 | 0 | 1 | 1 | 1 | 10 |
| van der Maarel-Wierink et al., 2014 [42] | 1 | 1 | 0 | 1 | 1 | 1 | 0 | 1 | 0 | 1 | 1 | 1 | 1 | 0 | 1 | 1 | 1 | 1 | 1 | 1 | 16 |
| Vidal Casariego, 2020 [43] | 1 | 1 | 1 | 1 | 1 | 1 | 1 | 1 | 0 | 0 | 1 | 0 | 0 | 1 | 1 | 1 | 1 | 1 | 1 | 1 | 16 |
| Wham et al., 2017 [44] | 1 | 1 | 0 | 1 | 1 | 0 | 0 | 1 | 1 | 1 | 1 | 1 | 0 | 0 | 1 | 1 | 1 | 1 | 0 | 1 | 14 |
| Young and Durant-Jones, 1990 [45] | 1 | 1 | 0 | 1 | 1 | 0 | 0 | 1 | 0 | 1 | 0 | 1 | 0 | 0 | 1 | 1 | 0 | 1 | 0 | 0 | 10 |

1. Downes, M.J., et al., *Development of a critical appraisal tool to assess the quality of cross-sectional studies (AXIS).* BMJ Open, 2016. **6**(12): p. e011458.
2. Abubakar, S.A. and B.Y. Jamoh, *Dysphagia following acute stroke and its effect on short-term outcome.* Niger Postgrad Med J, 2017. **24**(3): p. 182-186.
3. Andrade, P.A., et al., *The importance of dysphagia screening and nutritional assessment in hospitalized patients.* Einstein (Sao Paulo), 2018. **16**(2): p. eAO4189.
4. Arnold, M., et al., *Dysphagia in Acute Stroke: Incidence, Burden and Impact on Clinical Outcome.* PLoS One, 2016. **11**(2): p. e0148424.
5. Baroni, A.F., S.R. Fabio, and R.O. Dantas, *Risk factors for swallowing dysfunction in stroke patients.* Arq Gastroenterol, 2012. **49**(2): p. 118-24.
6. Beharry, A., et al., *Predictive Factors of Swallowing Disorders and Bronchopneumonia in Acute Ischemic Stroke.* J Stroke Cerebrovasc Dis, 2019. **28**(8): p. 2148-2154.
7. Blanař, V., et al., *Dysphagia and factors associated with malnutrition risk: A 5-year multicentre study.* J Adv Nurs, 2019. **75**(12): p. 3566-3576.
8. Brogan, E., et al., *Respiratory infections in acute stroke: nasogastric tubes and immobility are stronger predictors than dysphagia.* Dysphagia, 2014. **29**(3): p. 340-5.
9. Brogan, E., et al., *Dysphagia and factors associated with respiratory infections in the first week post stroke.* Neuroepidemiology, 2014. **43**(2): p. 140-4.
10. Carrión, S., et al., *Oropharyngeal dysphagia is a prevalent risk factor for malnutrition in a cohort of older patients admitted with an acute disease to a general hospital.* Clin Nutr, 2015. **34**(3): p. 436-42.
11. Chen, S., et al., *Prevalence and risk factors of dysphagia among nursing home residents in eastern China: a cross-sectional study.* BMC Geriatr, 2020. **20**(1): p. 352.
12. Crary, M.A., et al., *Dysphagia, nutrition, and hydration in ischemic stroke patients at admission and discharge from acute care.* Dysphagia, 2013. **28**(1): p. 69-76.
13. De Cock, E., et al., *Dysphagia, dysarthria and aphasia following a first acute ischaemic stroke: incidence and associated factors.* Eur J Neurol, 2020. **27**(10): p. 2014-2021.
14. Diendéré, J., et al., *Changes in nutritional state and dysphagia in stroke patients monitored during a 14-d period in a Burkina Faso hospital setting.* Nutrition, 2018. **48**: p. 55-60.
15. Falsetti, P., et al., *Oropharyngeal dysphagia after stroke: incidence, diagnosis, and clinical predictors in patients admitted to a neurorehabilitation unit.* J Stroke Cerebrovasc Dis, 2009. **18**(5): p. 329-35.
16. Finestone, H.M., et al., *Malnutrition in stroke patients on the rehabilitation service and at follow-up: prevalence and predictors.* Arch Phys Med Rehabil, 1995. **76**(4): p. 310-6.
17. Flowers, H.L., et al., *The incidence, co-occurrence, and predictors of dysphagia, dysarthria, and aphasia after first-ever acute ischemic stroke.* J Commun Disord, 2013. **46**(3): p. 238-48.
18. Gordon, C., R.L. Hewer, and D.T. Wade, *Dysphagia in acute stroke.* Br Med J (Clin Res Ed), 1987. **295**(6595): p. 411-4.
19. Groher, M.E.a.B., R., *The Prevalence of Swallowing Disorders in Two Teaching Hospitals.* Dysphagia, 1986. **1**: p. 3-6.

20. Hollaar, V.R.Y., et al., *Nursing home-acquired pneumonia, dysphagia and associated diseases in nursing home residents: A retrospective, cross-sectional study.* Geriatr Nurs, 2017. **38**(5): p. 437-441.
21. Huppertz, V.A.L., et al., *Association between Oropharyngeal Dysphagia and Malnutrition in Dutch Nursing Home Residents: Results of the National Prevalence Measurement of Quality of Care.* J Nutr Health Aging, 2018. **22**(10): p. 1246-1252.
22. Hägglund, P., et al., *Swallowing dysfunction as risk factor for undernutrition in older people admitted to Swedish short-term care: a cross-sectional study.* Aging Clin Exp Res, 2019. **31**(1): p. 85-94.
23. Jørgensen, L.W., et al., *Interrater reliability of the Volume-Viscosity Swallow Test; screening for dysphagia among hospitalized elderly medical patients.* Clin Nutr ESPEN, 2017. **22**: p. 85-91.
24. Kampman, M.T., et al., *Full Implementation of Screening for Nutritional Risk and Dysphagia in an Acute Stroke Unit: A Clinical Audit.* Neurohospitalist, 2015. **5**(4): p. 205-211.
25. Kidd, D., et al., *The natural history and clinical consequences of aspiration in acute stroke.* QJM, 1995. **88**(6): p. 409-13.
26. Lindroos, E., et al., *Caregiver-reported swallowing difficulties, malnutrition, and mortality among older people in assisted living facilities.* J Nutr Health Aging, 2014. **18**(7): p. 718-22.
27. Mañas-Martinez, A.B., et al., *Association of positive screening for dysphagia with nutritional status and long-term mortality in hospitalized elderly patients.* Endocrinol Diabetes Nutr, 2018.
28. Mateos-Nozal, J., et al., *High Prevalence of Oropharyngeal Dysphagia in Acutely Hospitalized Patients Aged 80 Years and Older.* J Am Med Dir Assoc, 2020. **21**(12): p. 2008-2011.
29. Melgaard, D., M. Rodrigo-Domingo, and M.M. Mørch, *The Prevalence of Oropharyngeal Dysphagia in Acute Geriatric Patients.* Geriatrics (Basel), 2018. **3**(2).
30. Nielsen, M.M., et al., *Associations between eating difficulties, nutritional status and activity of daily living in acute geriatric patients.* Clin Nutr ESPEN, 2018. **25**: p. 95-99.
31. Nogueira, D. and E. Reis, *Swallowing disorders in nursing home residents: how can the problem be explained?* Clin Interv Aging, 2013. **8**: p. 221-7.
32. Paciaroni, M., et al., *Dysphagia following Stroke.* Eur Neurol, 2004. **51**(3): p. 162-7.
33. Park, Y.H., et al., *Prevalence and associated factors of dysphagia in nursing home residents.* Geriatr Nurs, 2013. **34**(3): p. 212-7.
34. Patel, M.D. and F.C. Martin, *Why don't elderly hospital inpatients eat adequately?* J Nutr Health Aging, 2008. **12**(4): p. 227-31.
35. Rofes, L., et al., *Prevalence, risk factors and complications of oropharyngeal dysphagia in stroke patients: A cohort study.* Neurogastroenterol Motil, 2018: p. e13338.
36. Rösler, A., et al., *Dysphagia in Dementia: Influence of Dementia Severity and Food Texture on the Prevalence of Aspiration and Latency to Swallow in Hospitalized Geriatric Patients.* J Am Med Dir Assoc, 2015. **16**(8): p. 697-701.
37. Sarabia-Cobo, C.M., et al., *The incidence and prognostic implications of dysphagia in elderly patients institutionalized: A multicenter study in Spain.* Appl Nurs Res, 2016. **30**: p. e6-9.
38. Spronk, P.E., et al., *Prevalence and characterization of dysphagia in hospitalized patients.* Neurogastroenterol Motil, 2020. **32**(3): p. e13763.

39. Stipancic, K.L., et al., *Prospective Investigation of Incidence and Co-Occurrence of Dysphagia, Dysarthria, and Aphasia Following Ischemic Stroke.* Am J Speech Lang Pathol, 2019. **28**(1): p. 188-194.
40. Sugiyama, M., et al., *National survey of the prevalence of swallowing difficulty and tube feeding use as well as implementation of swallowing evaluation in long-term care settings in Japan.* Geriatr Gerontol Int, 2014. **14**(3): p. 577-81.
41. Tanigör, G. and S. Eyigör, *Evaluation of dysphagia in patients with sarcopenia in a rehabilitation setting: insights from the vicious cycle.* Eur Geriatr Med, 2020. **11**(2): p. 333-340.
42. van der Maarel-Wierink, C.D., et al., *Subjective dysphagia in older care home residents: a cross-sectional, multi-centre point prevalence measurement.* Int J Nurs Stud, 2014. **51**(6): p. 875-81.
43. Vidal Casariego, A., et al., *Utility of the EAT-10 in the detection of dysphagia in high-risk hospitalisation units at a university hospital: a cross-sectional study.* Nutr Hosp, 2020. **37**(6): p. 1197-1200.
44. Wham, C., et al., *Malnutrition risk of older people across district health board community, hospital and residential care settings in New Zealand.* Australas J Ageing, 2017. **36**(3): p. 205-211.
45. Young, E.C. and L. Durant-Jones, *Developing a dysphagia program in an acute care hospital: a needs assessment.* Dysphagia, 1990. **5**(3): p. 159-65.
